# Evaluating sex and gender as separate and interactive predictors of memory aging trajectory classes: an integrative data-driven approach

**DOI:** 10.1186/s13293-026-00864-2

**Published:** 2026-03-09

**Authors:** Linzy Bohn, Paige Vandenberg, Harrison Fah, M. Natasha Rajah, Gillian Einstein, Roger A. Dixon

**Affiliations:** 1 Department of Psychology, University of Alberta, P-217 Biological Sciences Building, Edmonton, T6G 2E9 AB Canada; 2 Neuroscience and Mental Health Institute, University of Alberta, Edmonton, AB Canada; 3 Department of Computing Science, University of Alberta, Edmonton, AB Canada; 4 Department of Psychology, Toronto Metropolitan University, Toronto, ON Canada; 5 Department of Psychiatry, Douglas Hospital Research Centre, McGill University, Montreal, QC Canada; 6 Department of Psychology, University of Toronto, Toronto, ON Canada; 7 Rotman Research Institute, Baycrest Hospital, Toronto, ON Canada

**Keywords:** Memory trajectories, Risk and protective factors, Sex, Gender, Latent class growth analysis, Random forest analysis, Cognitively unimpaired aging, Victoria Longitudinal Study, Apolipoprotein E

## Abstract

**Background:**

A priority in aging and dementia research is to integrate sex (biological attribute) and gender (sociocultural/behavioural characteristics) in theories, designs, analyses, and intervention protocols. We recently reported a data-mining procedure for operationalizing empirically-derived composite gender variables in archival databases. The present study extends the prior cross-sectional approach by examining sex and gender as separate and interactive predictors of longitudinal data-driven memory trajectory classes.

**Methods:**

Participants (*N =* 746) contributed baseline data for binary sex (female/male), education (years), and empirically-derived composite variables representing complementary gender facets. These facets included *Manual Tasks and Physical Activities; Social and Household Management*; *Leisure*,* Socializing*,* and Travel; Cognitive Activity and Brain Games*; *Health Perceptions and Practices*; and *Subjective Memory Beliefs*. We integrated these into a longitudinal episodic memory trajectory distribution spanning 42 years (53–95) of aging. Data-driven latent class growth analysis (LCGA) on the trajectory distribution identified discriminable classes. Using the R3STEP approach, we separately tested sex, gender facets, and education as predictors of membership in the higher (healthier) trajectory classes relative to the lowest (benchmark) class. We then included interaction terms to test for sex moderation of gender effects. Finally, we identified all genotyped participants and tested whether sex and gender effects were moderated by *Apolipoprotein E* (*APOE*).

**Results:**

LCGA revealed three memory classes: *High-Stable* (highest level/relatively stable), *Moderate/Normal-Declining* (average level/moderate decline), and *Low-Declining* (lowest level/steepest decline). Several variables separately predicted High-Stable membership. For sex, females were more likely than males to belong to this class. For gender, (a) higher scores for *Social and Household Management*, *Cognitive Activity and Brain Games*, and *Subjective Memory Beliefs* predicted High-Stable membership; and (b) higher scores for *Manual Tasks and Physical Activities* and *Health Perceptions and Practices* decreased the likelihood of High-Stable membership (relative to Low-Declining). Moderate/Normal-Declining membership was predicted by *Social and Household Management* (higher). For education, more years predicted High-Stable membership. Moderation analyses indicated that gender effects were consistent across both sexes and *APOE* carrier status.

**Conclusions:**

Data-driven analyses show that biological sex and measurable facets of gender differentially contribute to memory trajectory patterns over a 42-year span of cognitively unimpaired aging.

**Supplementary Information:**

The online version contains supplementary material available at 10.1186/s13293-026-00864-2.

## Abstract (plain language summary)

**The question we studied:**

We can learn about aging and dementia by assessing how the same persons change over multiple time points in life. We were interested in changes in memory abilities, as these are related to both healthy aging and dementia. Specifically, we compared how memory abilities of females and males changed over many years of aging and what experiences contributed to similar or different patterns.

**How we studied it:**

We studied a group of over 700 adults between the ages of 53 and 95. We explored how long-term changes in memory abilities were related to several personal characteristics, such as sex, gender, lifetime education, and genetic risk for Alzheimer’s disease.

**What we found:**

Our first finding was that there was a group that included both female and male adults who showed patterns of memory aging that remained high and stable into their 80s and 90s. Our second finding was that being a member of this higher group was more likely for females than males. Our third finding was that gender characteristics and education also played roles in predicting who was a member of this higher memory aging group.

**Why it matters:**

Our results help us to better understand how some experiences of aging females and males can lead to sustained patterns of healthier changes in memory abilities. More precise knowledge about how females and males differ in these patterns and their predictors is an important step in finding ways to promote healthier aging for both females and males.

**Supplementary Information:**

The online version contains supplementary material available at 10.1186/s13293-026-00864-2.

## Highlights

- Sex (biological attribute) and gender (sociocultural/behavioral characteristics) are distinct but related concepts that may separately or interactively contribute to differential memory aging. However, few datasets have broad coverage and differential measures representing sex *and *gender
- Data-driven analytics characterized 42-year changes in memory aging performance (53–95); tested whether memory change patterns are affected by both sex and gender; and determined whether such effects are similar for carriers and non-carriers of the leading Alzheimer’s genetic risk factor: *Apolipoprotein E *(*APOE*)
- We identified three discriminable subgroups of memory aging patterns, one of which was characterized by relatively higher performance levels and stable slopes into later life
- Membership in the High-Stable subgroup was predicted by biological sex (higher for females); multiple facets of gender, including lifestyle, perspectives, roles, and activities; and education (higher). This pattern was consistent across *APOE *carrier status
- Gendered risk/protection effects were consistent across sex

**Supplementary Information:**

The online version contains supplementary material available at 10.1186/s13293-026-00864-2.

## Background

A priority goal in aging and dementia research is to conceptually and operationally distinguish sex and gender and subsequently integrate each of these constructs into theories, designs, analyses, interpretations, and interventions [1–10]. For research applications, sex and gender can each be defined by separate but complementary (and potentially interactive) multi-dimensional domains of characteristics and representative variables [11–13]. Sex is commonly characterized by a heterogeneous set of biological and physiological attributes at birth (chromosomes, genes, gonads, hormones, and reproductive/sexual anatomy) that characterize and differentiate male, female, and intersex persons [1, 8, 11, 14, 15]. In contrast, gender is characterized by a comprehensive set of psychological, social, behavioral, and cultural characteristics that influence the explicit rules and implicit practices of identity, family, workplace, social-economic status, institutions, behaviours, expressions, norms, and roles that vary across men, women, and gender-diverse persons [1, 4, 11, 12, 14, 15].

Accordingly, aspects of either (or both) sex and gender may contribute to observed pathways and outcomes of brain and cognitive aging leading toward or away from dementia [3, 5, 15, 16]. Two prevailing challenges for empirically evaluating the relative contributions of variables reflecting the construct domains of sex and gender to differential patterns include: (a) many epidemiological and longitudinal databases operationalize sex and/or gender with a single binary self-report item and (b) few databases include pre-designed or direct gender measures, especially as related to aging and dementia. Recently [10], we reported a procedure for applying a novel data-mining approach to a large-scale database of human aging in order to operationalize empirically-derived composite variables representing complementary facets of gender. This archival database is characterized by the typical unavailability of measures reflecting multiple features and expressions of gender. Notably, however, the database is equipped with the compensatory strengths of broad and deep coverage of multiple gender-related domains. Our procedure was guided by the Gender Outcomes International Group: To Further Well-being Development (GOING-FWD) framework (for details see [11, 17]). This framework characterizes standardized methodological considerations for identifying, extracting, and operationalizing indirect gender variables in retrospective databases and incorporating them in empirical research.

Our systematic data-mining procedure revealed a diverse roster of candidate gender items related to everyday lifestyle and activities. These items were then reduced and assimilated—via data-driven principal component analysis (PCA)—into a meaningful set of six composite variables representing multiple facets of gender aging. The following interpretive labels were assigned (see Table 1 for constituent items): *Manual Tasks and Physical Activities (n* items = 6); *Social and Household Management (n* items = 6); *Leisure*,* Socializing*,* and Travel (n* items = 6); *Cognitive Activity and Brain Games (n* items = 5); *Health Perceptions and Practices (n* items = 6); and *Subjective Memory Beliefs (n* items = 8). We subsequently evaluated these six gender facets and education—an important indirect gender variable [3, 5, 18, 19]—as potential mediators of sex differences in cross-sectional episodic memory performance. Our results showed that older females performed better than males in episodic memory. These differences were mediated in part by selected gender facets, including everyday pursuits associated with Manual Tasks and Physical Activities, Social and Household Management, and Cognitive Activity and Brain Games. The often-studied lifetime education variable (number of years) also mediated the sex-memory association.

**Table 1:** Constituent Items for the Six Gender Facets (Principal Component Variables) and Corresponding Response Scales

Gender facet	Constituent gender-related items
Manual Tasks and Physical Activities (*n* items = 6)	Drive a car^a^ Do household repairs (painting, leaky faucets, etc.)^a^ Repair car, lawnmower, or other mechanical device^a^ Purchase new item requiring some set-up or assembly^a^ Woodworking, carpentry, or furniture refinishing^a^ Physical activity derived scale: Calculated by summing responses^a^ across four items: Gardening indoors or outdoors; Engage in exercise activities such as jogging, swimming, bicycling, or walking; Engage in outdoor activities such as sailing, fishing, or backpacking; Recreational sports such as tennis, bowling, or golf.
Social and Household Management (*n *items = 6)	Meal preparation^a^ Housework (dishes, laundry, vacuuming etc.)^a^ Food shopping^a^ Engage in sewing, knitting, or needlework^a^ Visit relatives, friends, or neighbours^a^ External memory strategy derived scale: Calculated by summing responses^b^ across 9 items: Do you keep a list or otherwise note important dates, such as birthdays and anniversaries; When you have not finished reading a book or magazine, do you somehow note the place where you stopped; Do you post reminders of things you need to do in a prominent place, such as bulletin boards or note boards; Do you routinely keep things in a familiar spot so you won’t forget them when you need to locate them; When you want to take something with you, do you leave it in an obvious, prominent place, such as putting your suitcase in front of the door; Do you ask other people to remind you of something; Do you write yourself reminder notes; Do you write appointments on a calendar to help you remember them; Do you write shopping lists.
Leisure, Socializing, and Travel (*n *items = 6)	Writing letters (to a friend, relative, business, etc.)^a^ Travel within province^a^ Travel outside of province (but within Canada)^a^ Travel in foreign country^a^ Give dinner party or party for friends^a^ Eat out at restaurants^a^
Cognitive Activity and Brain Games (*n *items = 5)	Crossword puzzles, acrostics, or anagrams^a^ Jigsaw puzzles^a^ Board games such as chess and checkers^a^ Knowledge games such as Trivial Pursuit^a^ Word games such as Scrabble^a^
Health Perceptions and Practices (*n *items = 6)	During the past four weeks, did sickness, injury, or health problem require you to visit the hospital^c^ During the past four weeks, did sickness, injury, or health problem require you to see a doctor (general practitioner or medical specialist)^c^ About how many times have you seen a doctor (GP or medical specialist) in the past year^d^ Compared to other people my age, I believe my overall health to be^e^ Compared to a perfect state of health, I believe my overall health to be^e^ Depression derived scale [118]: Calculated by summing responses^f^ across 20 items: I was bothered by things that don’t usually bother me; I did not feel like eating/my appetite was poor; I felt that I could not shake off the blues even with help from family; I felt that I was just as good as other people; I had trouble keeping my mind on what I was doing; I felt depressed; I felt that everything I did was an effort; I felt hopeful about the future; I thought my life had been a failure; I felt fearful; My sleep was restless; I was happy; I talked less than usual; I felt lonely; People were unfriendly; I enjoyed life; I had crying spells; I felt sad; I felt that people disliked me; I could not get “going”.
Subjective Memory Beliefs (*n *items = 8)	I am just as good at remembering as I ever was^g^ I can remember things as well as always^g^ I am good at remembering conversations I have had^g^ Memory anxiety derived scale: Calculated by summing responses^g^ across 14 items: I get upset when I cannot remember something; I find it harder to remember things when I am upset; I get anxious when I am asked to remember something; I have difficulty remembering things when I am anxious; I am usually uneasy when I attempt a problem that requires me to use my memory; I feel jittery if I have to introduce someone I just met; If I am put on the spot to remember names, I know I will have difficulty doing it; I would feel on edge if I had to take a memory test or something similar; When I am tense and uneasy at a social gathering I cannot remember names very well; When someone I don’t know very well asks me to remember something I get nervous; I get anxious when I have to do something I haven’t done for a long time; I get tense and anxious when I feel my memory is not as good as other people’s; I do not get flustered when I am put on the spot to remember new things; I would feel very anxious if I visited a new place and had to remember how to find my way back. I have little control over my memory ability^h^ I know if I keep using my memory I will never lose it^g^ I’m less efficient at remembering things now than I used to be^h^ Memory recruitment derived scale: Calculated by averaging responses^i^ across 5 items: When you want to remember an appointment do you ask somebody else (e.g., spouse of friend) to remind you; When an interesting T.V. program is going to be on in the next few days do you ask somebody else to help you remember (e.g., spouse or friend); When you want to remember the name of a particular person do you ask somebody else (e.g., spouse or friend) to help you remember; When you want to remember an event such as a birthday do you ask somebody else (e.g., spouse or friend) to help you remember; Do you sometimes ask people (e.g., spouse or friend) to help you remember when you are going to start a trip.

^a^ 0 = never; 1 = less than once a year; 2 = about once a year; 3 = 2 or 3 times a year; 4 = about once a month; 5 = 2 or 3 times a month; 6 = about once a week; 7 = 2 or 3 times a week; 8 = daily. ^b^1= never; 2 = rarely; 3 = sometimes; 4 = often; 5 = always. ^c^ 0 = no; 1= yes. ^d^ self-reported frequency. ^e^ 0 = very good; 0.25 = good; 0.50 = fair; 0.75 = poor; 1.00 = very poor. ^f^ 0 = rarely or none of the time; 1 = some or a little of the time (1–2 days), 2 = occasionally or a moderate amount of time (3–4 days); 3 = all of the time (5–7 days). ^g^ 1 = disagree strongly; 2 = disagree; 3 = undecided; 4 = agree; 5 = agree strongly. ^h^ 1 = agree strongly; 2 = agree; 3 = undecided; 4 = disagree; 5 = disagree strongly. ^i^ 1 = never, 2 = seldom, 3 = sometimes, 4 = often, 5 = always

Several large-scale longitudinal studies have evaluated sex for potential risk or protection effects on memory change trajectories and related clinical outcomes [3, 5, 15, 19–22]. Research conducted with cognitively unimpaired (or initially asymptomatic) older adults has produced a generally consistent pattern of results whereby female sex predicts higher and relatively stable patterns of memory performance, whereas male sex predicts comparatively lower levels and steeper decline [18, 23–28]. Accumulating literature shows that sex effects on memory aging may be moderated by a range of biologically-based factors [5, 29–31], such as neurological, neuroanatomical, gonadal, and hormonal characteristics. Genetic factors—particularly *Apolipoprotein E* (*APOE*)—have also been explored as potential contributors to sex differences in cognitively unimpaired memory aging [32–34], as well as incident risk for mild cognitive impairment (MCI), and Alzheimer’s disease (AD) [5, 18, 35–38]. A small but promising set of studies have indicated that sex may also interact with gender variables in predicting differential memory aging trajectories [3, 5, 19, 24, 39]. Much of this prior work has focused on the potential moderating role of single variables or domains, such as education, occupational complexity, and physical- or leisure-related activities. The present study extends this line of investigation in two ways. First, we evaluated whether membership in longitudinal data-driven episodic memory trajectory classes was separately predicted by sex, a broad range of empirically-derived gender facets [10], and education. Second, we tested whether gender facet and education effects were moderated by biological sex and *APOE*.

**Fig. 1:**
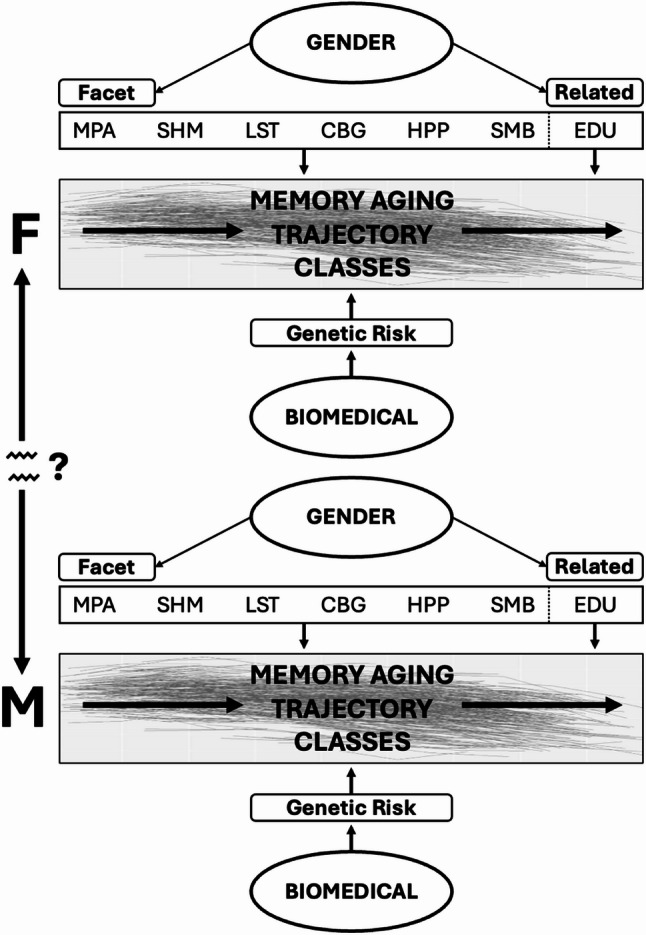
Empirically Evaluating Sex and Gender as Separable but Complementary Contributors to Differential Memory Aging. This figure provides a schematic representation of how sex and gender can be modeled as empirically distinguishable yet interrelated constructs that may contribute to differential memory aging trajectories. In the present study, gender was operationalized as a multi-faceted construct represented by six orthogonal principal component variables, as well as gender-related lifetime education (in years). Sex was represented by a single binary variable corresponding to biological categorizations of male and female. Our analytic workflow evaluated sex and gender as statistically separable predictors of membership in three data-driven trajectory classes, each of which represented a distinct pattern of memory performance and decline. Specifically, we sequentially tested the separate effects of sex, gender facets, and education on trajectory class membership. We then tested whether gender facet and education effects were moderated by sex. The placement of the sex variable in this model indicates that (a) sex may separately affect memory trajectories and (b) sex may interact with gender, such that the direction and/or magnitude of gender effects differs for males as compared to females. We evaluated selected biomedical characteristics (i.e., AD genetic risk) for separate and potentially interactive associations with trajectory class membership. Future research could leverage this approach to integrate biological sex, sociocultural gender, and related biomedical characteristics (e.g., vascular risk) into aging and dementia research. Annotation: Empirically distinguishing and separately testing variables representing the construct domains of sex and gender reflects statistical, theoretical, and practical considerations, not a claim about real-world independence. Abbreviations: F, Female; M, Male; MPA, Manual Tasks and Physical Activities; SHM, Social and Household Management; LST, Leisure, Socializing and Travel; CBG, Cognitive Activity and Brain Games; HPP, Health Perceptions and Practices; SMB, Subjective Memory Beliefs; EDU, Education

Three research goals (RG) were specified. For RG1, we applied latent class growth analysis (LCGA) to a distribution of longitudinal episodic memory trajectories covering a 42-year band of aging (53–95 years). This data-driven quantitative modeling technique applies an algorithm of level (intercept) and change (slope) to individualized trajectory data in order to detect computationally discriminable classes [40]. Accordingly, the resulting classes may not only represent discriminable subtypes of memory aging, but also secondary phenotypes that are characterized by differential risk for future impairment or dementia [27, 32, 41, 42]. For example, membership in higher and relatively sustained memory trajectory classes may correspond to a reduced risk or protection from pathways leading toward impairment and dementia. For RG2, we sequentially tested sex, gender facets [10], and education as separate predictors of membership in the data-driven memory trajectory classes. For RG3, we sequentially evaluated whether sex moderated gender facet and education effects on trajectory class predictions. We subsequently tested whether *APOE* separately or interactively predicted trajectory class membership. A schematic representation of this analytic workflow is provided in Fig. 1. We are unaware of prior research that has empirically distinguished these interrelated constructs and then tested the resulting variables for separate and potentially interactive associations with membership in data-driven memory trajectory classes. Accordingly, these results advance understanding of (a) how biological (sex, AD genetic risk) and sociocultural (gender facets, education) characteristics may each contribute to differential memory aging and (b) specific facets of lifestyle gender aging that could be targeted for the precision promotion of healthier memory aging.

## Methods

### Participants

We assembled longitudinal data for all participants from the cohort that was represented in our previous cross-sectional report [10]. The source cohort for our earlier study included 912 aging adults from the Victoria Longitudinal Study (VLS). As described in the previous report [10], participants were excluded if they were missing data (a) for self-reported sex (*n =* 0); (b) for education or one or more of the candidate gender items submitted to PCA (*n* = 96); or (c) across all episodic memory measures used to compile the latent variable (*n* = 30). Additional exclusionary criteria included a self-reported history of the following clinical conditions or considerations: (a) dementia diagnosis (*n* = 0); (b) moderate or severe stroke (*n* = 8); (c) moderate or severe Parkinson’s disease *(n* = 4); (d) severe epilepsy (*n* = 1); (e) severe head injury (*n* = 14); (f) use of antipsychotic or psychotropic medication (*n* = 4); and (g) rated severity of drug or alcohol dependence (*n* = 8). Participants with clinically low Mini-Mental State Exam [43] scores were also excluded (≤ 24; *n* = 1). The final cohort included 746 cognitively unimpaired older adults. These participants constitute the analytic sample for the present longitudinal report (*M* age *=* 71.9, range = 53.2–95.3 years; 66.8% female). See Table 2 (top panel) for further group characteristics.

**Table 2:** Baseline Participant Characteristics and Descriptive Statistics

Variable	Total cohort (*N* = 746)	Males (*n* = 248)	Females (*n* = 498)
**Background characteristics**			
Age (years)	71.92 (8.99)	73.47 (8.35)	71.16 (9.21)^***^
Range	53.24–95.25	54.13–95.25	53.24–91.31
MMSE	28.55 (1.35)	28.35 (1.37)	28.65 (1.33)^**^
Range	24–30	24–30	24–30
Currently married *n*(%) ^a^	438 (59%)	216 (87%)	222 (45%)^***^
Non-Hispanic White	737 (98.8%)	245 (99.2%)	492 (98.8%)
Education (years)	15.16 (2.94)	15.78 (3.14)	14.85 (2.79)^***^
Range	5.0–23.5	8.0–23.0	5.0–23.5
*APOE* ε4 carrier ^b^	130	44 (24.6%)	86 (24.1%)
ε4H	10 (1.9%)	5 (2.8%)	5 (1.4%)
ε4h	120 (22.4%)	39 (21.8%)	81 (22.7%)
*APOE* ε2 carrier ^b^	71 (13.2%)	21 (11.7%)	50 (14%)
ε2H	33 (6.2%)	9 (5%)	24 (6.7%)
ε2h	38 (7.1%)	12 (6.7%)	26 (7.3%)
**Gender facets (principal component variables)**			
Manual Tasks and Physical Activities	0 (1.00)	0.78 (1.06)	−0.39 (0.70)^***^
Social and Household Management	0 (1.00)	−0.72 (1.09)	0.36 (0.72)^***^
Leisure, Socializing, and Travel	0 (1.00)	0.04 (1.02)	−0.02 (0.99)
Cognitive Activity and Brain Games	0 (1.00)	−0.15 (0.98)	0.07 (1.00)^**^
Heath Perceptions and Practices	0 (1.00)	−0.09 (0.93)	0.04 (1.03)^^^
Subjective Memory Beliefs	0 (1.00)	−0.09 (1.04)	0.05 (0.98)^^^

Results presented as mean (standard deviation) unless specified as otherwise. *p-*values are based on independent samples *t-*test or chi-square test, as appropriate. MMSE, Mini Mental State Exam; *APOE*, *Apolipoprotein E*; ε4H, Homozygous; ε4h, Heterozygous; ε2H, Homozygous; ε2h, Heterozygous. ^a^ These values reflect the proportion of participants currently married. Across the total cohort, 36 (5%) were single, 156 (21%) were widowed, 107 (14%) were divorced, and 9 (1%) were separated. For males: 4 (2%) were single, 10 (4%) were widowed, 14 (5%) were divorced, and 4 (2%) were separated. For females: 32 (6%) were single, 146 (29%) were widowed, 93 (19%) were divorced, and 5 (1%) were separated. ^b^ A subset of participants were genotyped (*n =* 562). Carriers of the ε2ε4 genotype (*n* = 26) were excluded from all R3STEP *APOE* analyses (final *n* = 536; *n* females = 357 (66.6%); *M* age *=* 70.9, range = 53.2–95.3 years)

^***^ *p*-value < 0.001 ^**^ *p*-value < 0.01 ^^^ *p-*value < 0.10

Data collection procedures in the VLS were in full compliance with prevailing research ethics guidelines and approved by the Human Research Ethics Board. All participants provided written and informed consent. Participant retention rates for the two adjoining timepoints were 70% and 74%. Continuing participants were tested at an average interval of 4.4 years. As noted above, our established cohort [10] had no missing data for the baseline predictors of (a) self-reported sex, (b) component scores for the six empirically-derived gender facets, and (c) education. No memory data were missing at the first timepoint. Missing memory data at the second and third timepoints were assumed to be missing at random and were estimated in all corresponding analyses using full information maximum likelihood [42, 44–46].

### Measures

#### Sex

The VLS measures biological sex by asking participants to self-report as either male or female. At the time of data collection, this binary approach was the standard for large-scale aging databases and has been employed in numerous related works [4, 10, 24, 27, 34, 47, 48].

#### Empirically-derived gender facets

We previously [10] adapted the GOING-FWD framework [11, 17] to the multi-domain VLS archives in order to identify, extract, and operationalize indirect gender variables. We summarize our application of this adapted framework. For details, see our earlier report [10]. The GOING-FWD framework uses four gender dimensions proposed by the Women’s Health Research Network [11] to operationalize and conceptualize indirect gender variables, including: *Gender Identity*, *Gender Roles*, *Gender Relations*, and *Institutionalized Gender*. *Gender Identity* refers to an individual’s personal identification of themselves along a continuum of man, woman, or other. *Gender Roles* refer to the behavior learned by a person as appropriate to their gender, determined by the prevailing cultural norms (e.g., status of household’s primary responsibilities). *Gender Relations* refers to interactions and relationships between individuals based on gender (e.g., social support). *Institutionalized Gender* refers to a domain within a population that could present gender discrepancies (e.g., policies on family leave). In our previous report [10], we operationalized these conceptual definitions and implemented them in the multifaceted VLS data archives. The aim was to identify candidate items representing each of the four gender dimensions. Our systematic and theory-guided approach yielded an initial heterogeneous roster of 56 items that corresponded to three of the four gender dimensions represented in the GOING-FWD framework, viz., Gender Roles, Gender Relations, and Institutionalized Gender. We subsequently applied PCA to the full set of 56 items [10] in order to empirically identify a heterogenous set of multi-indicator component variables that collectively represent distinct (or computationally separable) facets of lifestyle gender aging. Findings revealed six principal component variables, each of which featured multiple items (*N* = 37; Table 1). Interpretative labels were assigned to gender facets based on empirical separation and common characteristics (Supplementary Table 1, Additional File 1) of the constituent items (Table 1).

We summarize the obtained and interpreted gender facets. *Manual Tasks and Physical Activities* reflects participation in manual maintenance household tasks and physical activities (domestic and non-domestic). *Social and Household Management* reflects engagement in social, instrumental, and managerial domestic activities. *Leisure*,* Socializing*,* and Travel* reflects participation in activities undertaken for enjoyment or well-being. *Cognitive Activity and Brain Games* represents engagement in cognitively stimulating activities. *Health Perceptions and Practices* represents subjective evaluations of physical and emotional health, as well as levels of engagement in related health-seeking behaviors. *Subjective Memory Beliefs* reflects one’s conceptions of day-to-day memory performance. We assembled baseline principal component scores for each of these gender facets (see Table 2 (lower panel) for descriptive statistics). Higher component scores for the following facets denote greater levels of engagement in the associated pursuits: Manual Tasks and Physical Activities; Social and Household Management; Leisure, Socializing, and Travel; and Cognitive Activity and Brain Games. Higher component scores for Health Perceptions and Practices denotes more negative conceptions of one’s physical and emotional health, as well as increased healthcare engagement. Higher component scores for Subjective Memory Beliefs denotes more positive conceptions of one’s memory ability.

#### Education

Education has been studied as gender-related for this cohort of aging adults [10], as well as in related large-scale longitudinal studies [7, 49, 50]. Consistent with previous research [10, 27, 32], we used the baseline self-report of the highest number of years of formal schooling (see Table 2 (top panel) for descriptive statistics).

#### *APOE*

From the current cohort, we identified all participants (*n* = 562) who had contributed saliva to the VLS. These participants had been genotyped according to standard biofluid collection, stabilization, and preparation procedures from Oragene DNA Genotek technology (for details see [34]). Based on prior research indicating that the ε4 allele represents a risk factor [32, 33, 36, 38] and the ε2 allele represents a protection factor [34, 51–54], we excluded participants with ε2ε4 genotype (*n* = 26) from all reported *APOE* analyses. Accordingly, the remaining genotyped participants included 536 older adults (*M* age *=* 70.9, range = 53.2–95.3 years; 66.6% female) with characteristics closely approximating the original cohort (i.e., *M* age *=* 71.9, range = 53.2–95.3 years; 66.8% female). We clustered the *APOE* genotype into two dichotomous classifications (see Table 2 for allelic frequencies): (a) ε4- (ε2/ε2, ε2/ε3, ε3/ε3) vs. ε4+ (ε3/ε4, ε4/ε4); and (b) ε2- (ε3/ε3, ε3/ε4, ε4/ε4) vs. ε2+ (ε2/ε2, ε2/ε3). The genotypic distribution for the ε4 allele conformed to Hardy-Weinberg equilibrium (χ²_(1)_ = 0.04, *p* = .85), whereas the ε2 distribution did not (χ²_(1)_ = 137.02, *p* <.001). The latter result was primarily driven by a higher-than-expected number of ε2/ε2 homozygotes (see Table 2). This pattern has been observed in other volunteer-based studies (e.g. [55]), and is not attributable to intentional oversampling of the ε2 allele in the VLS.

#### Memory

Three longitudinal verbal episodic memory performance measures were used to compute a single-factor latent variable [10, 27, 48] at each timepoint (using Mplus 8.10 [56]). As described in the Supplementary Methods (Additional File 1), the measures included: (a) the VLS Word Recall [57] and (b) two list tasks from the Rey Auditory Verbal Learning Test [58, 59]. We selected the latent variable approach (over manifest single or composite variable approaches) based on the considerations that such variables (a) establish the content, criterion, and construct validity of the latent variable under investigation and (b) correct for sources of measurement error that affect the reliability of the latent construct [46].

### Analytical approach

#### Statistical characteristics of the predictor variables

We examined descriptive (sex) differences between male and female participants in mean principal component scores for the six gender facets, as well as education, using independent-samples *t*-tests.

#### Foundational analyses for the latent memory variable

We tested and confirmed key measurement characteristics of the latent memory variable as follows (Mplus 8.10 [56]). First, we applied confirmatory factor analysis to baseline data for the three memory measures. Model fit was determined using the following standard indices [10, 46]: (a) χ^2^, for which a non-significant result indicates good fit; (b) comparative fit index (CFI), for which a value of ≥ 0.95 indicates good fit and ≥ 0.90 adequate fit; (c) root mean square error of approximation (RMSEA), for which a value of ≤ 0.05 indicates good fit and ≤ 0.08 adequate fit; and (d) standardized root mean square residual (SRMR), for which a value of ≤ 0.08 indicates good fit.

Second, we tested longitudinal measurement of invariance of the latent memory variable by evaluating (a) configural invariance, which stipulates the same pattern of factor loadings over the three timepoints; (b) metric invariance, which stipulates the same magnitude of factor loadings over time; and (c) scalar invariance, which stipulates that the intercepts of the memory measures are the same over time. The tenability of invariance assumptions was tested by comparing models with unconstrained and constrained parameters using change in CFI (*≤* 0.01 suggests that the assumption is reasonable [46, 60]). For each participant, we estimated factor scores (i.e., standardized values; the higher the better) on the latent memory variable using the best-fitting, constrained model. These data were employed in all subsequent memory-related analyses.

Third, we established the functional form of memory change by testing the following sequence of linear, unconditional latent growth models: (a) fixed intercept, which assumes no intra- or inter-individual variability in memory performance; (b) random intercept, which allows for inter-individual differences in memory performance but assumes no intra-individual change; (c) random intercept/fixed slope, which allows for inter-individual differences in memory performance but assumes that each person changes at the same rate; and (d) random intercept/random slope, which allows for inter-individual differences in memory performance and change [61]. Consistent with prior work [27, 41, 42], actual chronological age was used as the metric of memory change. This approach is preferred over wave-based analyses as it (a) accounts for the effects of age as though it were a study covariate and (b) improves interpretability of the study findings [62]. Age was centered at 75 years, which is the approximate mean of the 42-year band of memory trajectory data and a common inflection point in brain and cognitive aging [63].

#### Identifying distinguishable classes of memory aging trajectories

LCGA was used to detect classes representing differential patterns of memory aging trajectories (RG1). This data-driven approach implements an algorithm based on intercept and slope to extract latent classes from the overall distribution of memory trajectories. We fit a sequence of six linear models with a varying number of trajectory *k-*classes (e.g., *k* = 1, 2, 3). An assumption of each *k*-class model is that there is no within-class variability in the growth parameters (intercept, slope). Each model was run with 500 random starting values in order to avoid local maxima. We selected the best fitting *k*-class model based on three observed characteristics of the latent memory classes (parsimony, interpretability, relevant literature), as well as the following model parameters, tests, and relative fit indices: (a) log-likelihood value (LL), (b) number of parameters estimated, (c) Bayesian Information Criterion (BIC), (d) sample-size adjusted BIC (SABIC), and (e) Akaike Information Criterion (AIC). Lower values of BIC, SABIC, and AIC indicate better fit. Entropy is not used for model selection but instead (a) suggests the accuracy with which participants are classified into the trajectory classes (ranges between 0 and 1; higher means better) and (b) serves as a robust indicator of the degree of separation between trajectory classes (higher means better).

#### Predicting trajectory class membership

We evaluated the baseline predictors for separate (RG2) and potentially interactive (RG3) effects on trajectory class membership using bivariable and multivariable multinomial logistic regression, respectively. We performed these analyses using the R3STEP approach (for details see [64]). Two advantages of this approach are that (a) it ensures that the formation and structure of the latent trajectory classes is unaffected by the inclusion of predictor variables and (b) it correctly treats trajectory class membership as a latent (in contrast to manifest) variable, thereby enabling classification uncertainty to be taken into account. The first step of our R3STEP prediction analyses involved sequentially testing sex, gender facets, and gender-related lifetime education as separate predictors of trajectory class membership (RG2). The resulting odds ratios (OR) represent the ratio of the odds of membership in a target trajectory class (i.e., High-Stable, Moderate/Normal-Declining) relative to the odds of membership in a common benchmark class (i.e., Low-Declining). The second step of our R3STEP prediction analyses involved (a) separately testing whether each successive gender facet and education interacted with sex (RG3) and then (b) separately testing whether sex, each successive gender facet, and education interacted with *APOE*. The procedure for moderation analyses was three-fold. First, any statistically significant interaction terms were probed by conducting (a) sex-stratified analyses (i.e., evaluating prediction effects separately for males and females) or (b) *APOE*-stratified models, as appropriate. Second, non-significant interaction terms were (a) interpreted as representing that the direction and magnitude of the corresponding predictor did not statistically differ for males as compared to females and (b) dropped from the corresponding model (for parsimony and interpretability). Third, the corresponding multivariable R3STEP model was re-run. The latter approach estimates the conditional direct effect of sex and the jointly examined gender variable.

## Results

### Statistical characteristics of the predictor variables

As displayed in Table 2, male and female sexed participants differed significantly in mean principal component scores for three gender facets, viz., Manual Tasks and Physical Activities (higher average participation for males); Social and Household Management (higher average participation for females); and Cognitive Activity and Brain Games (higher average participation for females). Three gender facets were not characterized by statistically significant sex differences in mean principal component scores, viz., Leisure, Socializing, and Travel; Health Perceptions and Practices; and Subjective Memory Beliefs. Regarding gender-related lifetime education, males had more average years of formal schooling relative to females. In Supplementary Table 2 (Additional File 1), we quantify both the direction and magnitude of associations among sex, gender facets, and education using point-biserial or Pearson correlation coefficients, as appropriate.

### Foundational analyses for the latent memory variable

First, results from the confirmatory factor analysis verified that a single-factor latent variable model fit the data well (Supplementary Table 3, Additional File 1). Second, longitudinal measurement invariance tests of the latent memory variable indicated full metric and full scalar invariance (Supplementary Table 3, Additional File 1; final model fit indices: CFI = 0.98; RMSEA = 0.06; SRMR = 0.06). Third, findings from the latent growth models showed that a random intercept/random slope model fit the data best (Supplementary Table 4, Additional File 1). The latter findings indicate that participants demonstrated (a) significant variability in their level of memory performance at the centering age of 75 ($\:\widehat{{\upsigma\:}}$^2^ = 0.90, *p* <.001; *M*_intercept_ = −0.30, *p* <.001), (b) significant decline over time (*M*_slope_ = −0.09, *p* <.001), and (c) significant inter-individual differences in the rate of decline ($\:\widehat{{\upsigma\:}}$^2^ = 0.001, *p* <.001). Collectively, these findings provide empirical support for evaluating classes of memory aging trajectories using LCGA.

### Identifying distinguishable classes of memory aging trajectories

We report model parameters, tests, and relative fit indices from the LCGA in Table 3 (RG1). The three-class memory trajectory model was selected as the optimal solution based on four considerations. First, as reported in Table 3, the three-class model was characterized by lower AIC, BIC, and SABIC values as compared to the one- and two-class models. In addition, the primary inflection point (or “elbow”) in the distribution of these relative fit indices occurs at the three-class solution (Supplementary Fig. 1, Additional File 1), indicating that model improvement tapers off for the four- through six-class solutions. Second, the three-class model was characterized by an entropy value of 0.83, which suggests that (a) participants were classified into the memory trajectory classes with a relatively high degree of certainty and (b) there is sufficient empirical separation between the three trajectory classes. Third, the prevalence of each class exceeded the standard criterion cut-off of > 10% [46]. Fourth, the intercept and slope parameters (Table 4) converged with those reported in previous trajectory analyses of memory [27, 42] and brain [41] aging.

**Table 3:** Model Fit Statistics for Unconditional Latent Class Growth Models of Memory

k-class model	(-)2LL	npar	AIC	BIC	SABIC	Entropy
1	4272.62	5	4282.62	4305.69	4289.82	--
2	3554.14	8	3570.15	3607.06	3581.66	0.75
**3**	**3083.60**	**11**	**3105.60**	**3156.36**	**3121.43**	**0.83**
4	2834.95	14	2862.95	2927.56	2883.10	0.83
5	2571.90	17	2605.90	2684.35	2630.37	0.84
6	2470.80	20	2510.80	2603.09	2539.58	0.85

LL, log-likelihood; npar, number of parameters free; AIC, Akaike information criterion; BIC, Bayesian information criterion; SABIC, sample-size adjusted BIC. The best fitting model is bolded

**Table 4:** Parameter Estimates for the Best-Fitting Unconditional Latent Class Growth Model of Memory (*k* = 3)

Parameter Estimates	Class 1: High-Stable (*n* = 260; 34.9%)	Class 2: Moderate/Normal-Declining (*n* = 349; 46.7%)	Class 3: Low-Declining (*n* = 137; 18.4%)
Intercept [95% CI]	0.62 [0.52, 0.73]	−0.44 [−0.59, −0.32]	−1.60 [−1.84, −1.43]
Slope [95% CI]	−0.05 [−0.06, −0.04]	−0.07 [−0.08, −0.07]	−0.08 [−0.09, −0.07]

CI, confidence interval

**Fig. 2:**
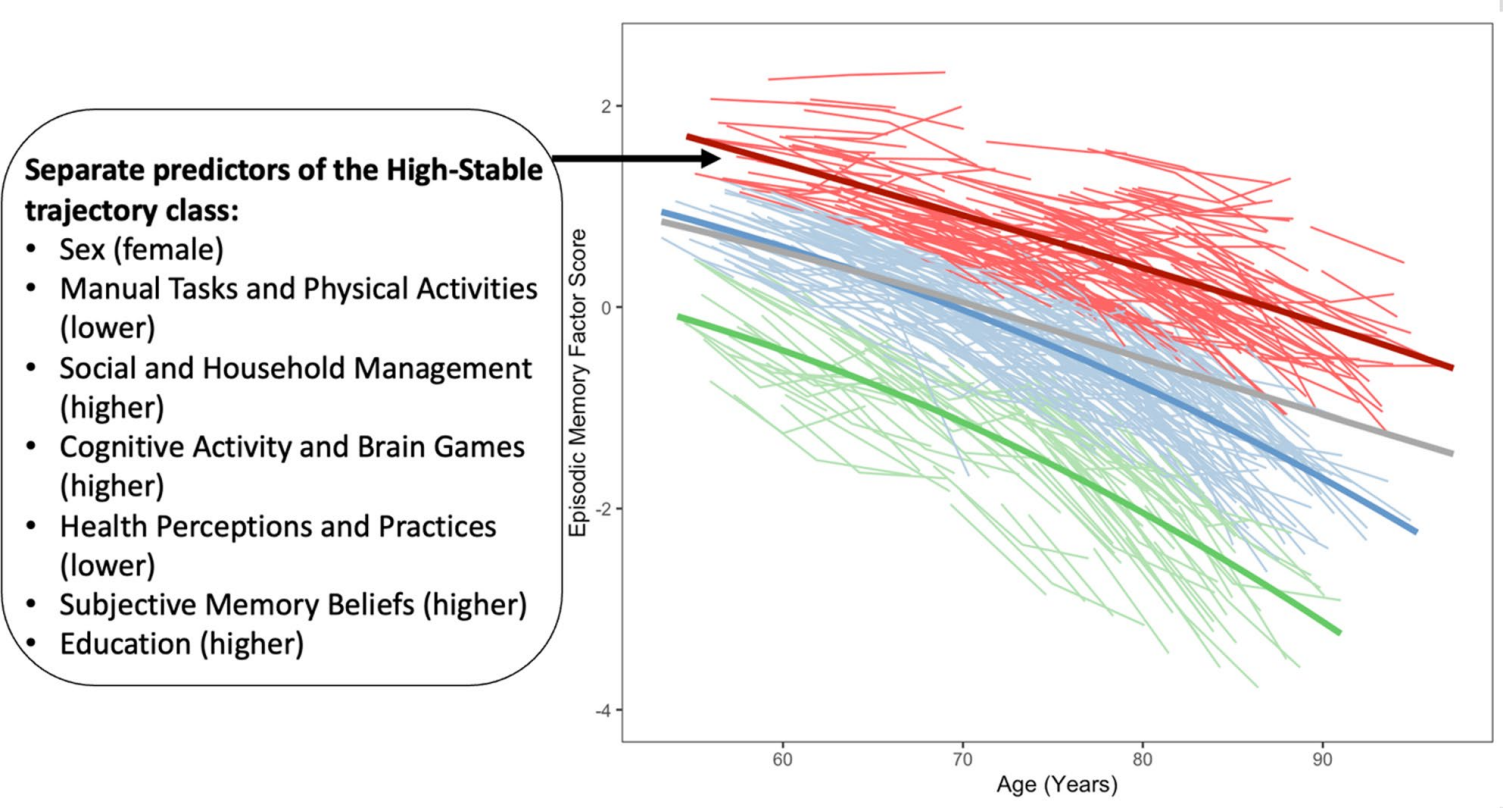
Individualized (raw) trajectories of episodic memory (color-coded for class membership). The following three latent trajectory classes were differentiated based on an algorithm of combined intercept and slope parameters: *High-Stable* (red; characterized by the highest level and relative maintenance over time), *Moderate/Normal-Declining* (blue; characterized by average level and moderate decline), and *Low-Declining* (green; characterized by the lowest level and steeper decline). The dark colored lines represent the average change trajectory for the corresponding class. The gray line represents the average change trajectory for the overall cohort. The text box summarizes results for RG2, in which we demonstrated that both sex and gender facets, as well as education, separately predicted the membership in the highest trajectory class (direction of effects is noted in parentheses). Membership in the Moderate/Normal-Declining class was selectively predicted by Social and Household Management (higher)

Trajectory distributions for the three memory classes are displayed in Fig. 2. We assigned the following interpretive labels based on prior work [27, 42] and observed class differences in combined intercept and slope parameters: *High-Stable* (*n* = 260, 34.9%), *Moderate/Normal-Declining* (*n* = 349, 46.7%), and *Low-Declining* (*n* = 137, 18.4%). As displayed in Fig. 2, the High-Stable class is characterized by above-average memory performance (intercept = 0.62; 95% confidence interval [CI]:  0.52, 0.73) and a relatively sustained trajectory over time (slope = −0.05; 95% CI: −0.06, −0.04). The Moderate/Normal-Declining class is characterized by mid-range memory performance (intercept = −0.44; 95% CI: −0.59, −0.32) and moderately steeper decline (slope = −0.07; 95% CI: −0.08, −0.07). The Low-Declining class is characterized by the lowest level (intercept = −1.60; 95% CI: −1.84, −1.43) and steepest decline (slope = −0.08; 95% CI: −0.09, −0.07).

### Predicting trajectory class membership

For all reported R3STEP prediction analyses, the Low-Declining trajectory class was specified as the common benchmark given that it is computationally and functionally most distinct from the other two classes. The resulting ORs represent the ratio of odds of membership in the High-Stable or Moderate/Normal-Declining trajectory class relative to the Low-Declining class. Statistical significance of prediction effects was inferred from the corresponding 95% CI [64–66].

Table 5 presents results from our first set of bivariable R3STEP prediction models (RG2), in which we sequentially evaluated the separate effect of each predictor on trajectory class membership. With respect to sex, females were 2.65 times more likely than males to belong to the High-Stable (OR = 2.65; 95% CI: 1.64, 4.30) relative to Low-Declining trajectory class. Next, we separately tested each of the six gender facets, as well as education, using the combined data from males and females (i.e., as collapsed across sex). Regarding gender facets, higher component scores for Social and Household Management (OR = 1.47; 95% CI: 1.16, 1.85), Cognitive Activity and Brain Games (OR = 1.79; 95%: 1.39, 2.30), and Subjective Memory Beliefs (OR = 1.31; 95%: 1.05, 1.63) increased the relative odds of High-Stable membership. Additionally, higher component scores for Manual Tasks and Physical Activities (OR = 0.76; 95% CI: 0.60, 0.95) and Health Perceptions and Practices (OR = 0.79; 95% CI: 0.63, 0.99) decreased the odds of membership in the High-Stable relative to Low-Declining trajectory class. The Moderate/Normal-Declining class was predicted only by Social and Household Management (OR = 1.22; 95% CI: 1.0, 1.48), whereby higher component scores increased the relative odds of membership. One gender facet—Leisure, Socializing, and Travel—did not significantly predict trajectory class membership. Regarding education, we found that more years of formal schooling increased the relative odds of High-Stable membership (OR = 1.10; 95% CI: 1.01, 1.19). We summarize these separate prediction effects in Fig. 2.

**Table 5:** Separate Effect of Sex, Gender Facets, and Education on Trajectory Class Membership (Reference = *Low-Declining*)

**Baseline predictor**	Trajectory Class	
	High-Stable	Moderate/Normal-Declining
	OR [95% CI]	OR [95% CI]
Self-reported sex (0 = male, 1 = female)	2.65 [1.64, 4.30] ^*^	1.43 [0.90, 2.28]
Manual Tasks and Physical Activities	0.76 [0.60, 0.95] ^*^	0.89 [0.72, 1.10]
Social and Household Management	1.47 [1.16, 1.85] ^*^	1.22 [1.0, 1.48] ^*^
Leisure, Socializing, and Travel	1.10 [0.88, 1.39]	1.03 [0.81, 1.30]
Cognitive Activity and Brain Games	1.79 [1.39, 2.30] ^*^	1.22 [0.95, 1.58]
Health Perceptions and Practices	0.79 [0.63, 0.99] ^*^	0.91 [0.72, 1.14]
Subjective Memory Beliefs	1.31 [1.05, 1.63] ^*^	1.11 [0.89, 1.39]
Education (in years)	1.10 [1.01, 1.19] ^*^	1.00 [0.92, 1.09]

OR, odds ratio; CI, confidence interval. Higher component scores for the following facets denote greater levels of engagement in the associated pursuits: Manual Tasks and Physical Activities; Social and Household Management; Leisure, Socializing, and Travel; and Cognitive Activity and Brain Games. Higher component scores for Health Perceptions and Practices denotes less positive subjective health (physical, emotional) and increased health-seeking behaviors. Higher component scores for Subjective Memory Beliefs denotes more positive memory conceptions.

^*^ Denotes statistically significant R3STEP prediction effects (based on a 95% CI that does not include 0)

In our second set of R3STEP prediction models (RG3), we sequentially tested whether sex moderated gender facet and education effects. Each multivariable model included main effect (*n* = 2) and interaction (*n* = 1) terms. Results showed that sex and gender did not interactively predict trajectory class membership (as indicated by null interaction terms; Supplementary Table 5, Additional File 1). Specifically, the direction and magnitude of gender facet and education effects did not statistically differ for males as compared to females. We tested and confirmed the robustness of this pattern using a complementary analytical approach, “classify-analyze”. In the first step, participants were classified into the memory trajectory classes using estimated posterior probabilities [65, 67]. In the second step, we used multinomial logistic regression with main effect (*n* = 2) and interaction (*n* = 1) terms to test for sex moderation of gender facet and education effects. Once again, all tested models were characterized by null interaction terms (Supplementary Table 6, Additional File 1).

Accordingly, we (a) dropped non-significant interaction terms from each successive R3STEP model and subsequently (b) conducted post-hoc tests in order to examine the relative impact of sex and each successive gender variable on trajectory class predictions. Specifically, we performed a series of multivariable R3STEP logistic regressions with two main effect terms. The corresponding results represent (a) the conditional direct effect of sex with each successive gender variable held constant (i.e., statistically covaried) and (b) the conditional direct effect of each gender variable with sex held constant. As shown in Table 6, robust prediction results were detected for the extreme class comparison (i.e., High-Stable vs. Low-Declining). Regarding sex, we found that across gender variable models, females were between 2 and 3 times more likely than males to belong to the High-Stable relative to Low-Declining trajectory class. Regarding gender facets, the following variables remained significant predictors of High-Stable membership when sex was statistically covaried: Cognitive Activity and Brain Games (OR = 1.74; 95% CI: 1.35, 2.24), Health Perceptions and Practices (OR = 0.77; 95% CI: 0.61, 0.97), and Subjective Memory Beliefs (OR = 1.28; 95% CI: 1.02, 1.60). Education also remained a significant predictor of High-Stable membership when tested alongside sex (OR = 1.13; 95% CI: 1.04, 1.23). In sum, these post-hoc tests indicated that, when variables representing the construct domains of sex and gender are tested in the same computational context, both contribute to differential memory trajectories.

**Table 6:** Conditional Direct Effects of Sex, Gender Facets, and Education on Trajectory Class Membership

	**Trajectory class**	
	*High-Stable*	*Moderate/Normal-Declining*
	OR [95% CI]	OR [95% CI]
***Model 1***		
Self-reported sex^a^	2.54 [1.43, 4.50]^*^	1.37 [0.79, 2.36]
Manual Tasks and Physical Activities	0.96 [0.73, 1.26]	0.96 [0.74, 1.24]
***Model 2***		
Self-reported sex^a^	2.13 [1.24, 3.67]^*^	1.19 [0.69, 2.03]
Social and Household Management	1.22 [0.95, 1.57]	1.18 [0.93, 1.50]
***Model 3***		
Self-reported sex^a^	2.67 [1.65, 4.34]^*^	1.43 [0.90, 2.28]
Leisure, Socializing, and Travel	1.12 [0.89, 1.41]	1.03 [0.82, 1.31]
***Model 4***		
Self-reported sex^a^	2.44 [1.50, 3.99]^*^	1.39 [0.87, 2.22]
Cognitive Activity and Brain Games	1.74 [1.35, 2.24]^*^	1.21 [0.93, 1.56]
***Model 5***		
Self-reported sex^a^	2.77 [1.70, 4.53]^*^	1.46 [0.91, 2.32]
Health Perceptions and Practices	0.77 [0.61, 0.97]^*^	0.89 [0.71, 1.23]
***Model 6***		
Self-reported sex^a^	2.58 [1.59, 4.20]^*^	1.41 [0.89, 2.25]
Subjective Memory Beliefs	1.28 [1.02, 1.60]^*^	1.10 [0.88, 1.37]
***Model 7***		
Self-reported sex^a^	3.04 [1.83, 5.05]^*^	1.45 [0.90, 2.33]
Education (years)	1.13 [1.04, 1.23]^*^	1.01 [0.93, 1.10]

OR, odds ratio; CI, confidence interval. ^a^Sex was categorized as 0 = male, 1 = female. For each R3STEP model, the *Low-Declining* class was specified as the common benchmark. Higher component scores for the following facets denote greater levels of engagement in the associated pursuits: Manual Tasks and Physical Activities; Social and Household Management; Leisure, Socializing, and Travel; and Cognitive Activity and Brain Games. Higher component scores for Health Perceptions and Practices denotes less positive subjective health (physical, mental) and increased health-seeking behaviors. Higher component scores for Subjective Memory Beliefs denotes more positive memory conceptions.

^*^Denotes statistically significant R3STEP prediction effects (based on a 95% CI that does not include 0)

#### Testing *APOE* as a predictor of trajectory class membership

Results from the bivariable R3STEP model indicated that ε4 allelic carrier status did not separately predict the relative odds of High-Stable (OR = 0.63; 95% CI: 0.34, 1.16) or Moderate/Normal-Declining membership (OR = 0.80; 95% CI: 0.44, 1.48). Similarly, ε2 carrier status did not separately predict the relative odds of High-Stable (OR = 2.25; 95% CI: 0.82, 6.20) or Moderate/Normal-Declining membership (OR = 2.37; 95% CI: 0.84, 6.67). Successive multivariable R3STEP moderation analyses revealed that sex, gender facet, and education effects did not vary according to *APOE* ε4 and ε2 carrier status. Null interaction terms for the respective models are reported in Supplementary Tables 7 and 8 (Additional File 1). Conditional direct effects of *APOE* ε4 and each successive predictor (sex, gender facets, education) are reported in Supplementary Table 9 (Additional File 1). Supplementary Table 10 (Additional File 1) reports conditional direct effects of *APOE* ε2 and each successive predictor. We tested and confirmed that *APOE* (ε4, ε2) did not separately or interactively predict trajectory class membership using the complementary “classify-analyze” approach.

#### Follow-up, data-driven exploratory analyses

By design, our R3STEP prediction analyses focused on providing novel insight into the separate (RG2) and potentially interactive (RG3) effects of biological sex and multiple facets of sociocultural gender on differential memory aging trajectories. Insights about comparative (or rank-ordered) prediction roles can be garnered from computationally competitive machine learning analyses. In a recent comparative review, we evaluated the computational characteristics and relative performance of such approaches for applications in aging and dementia research [68; see also 69 for an application]. Our results indicated that random forest analysis (RFA) and Tree Shapley Additive exPlanations (Tree SHAP) are robust and effective approaches for evaluating the relative importance of a battery of risk and protection factors to differential memory aging trajectories and related clinical outcomes. To date, no prior research has leveraged these approaches to advance understanding of the comparative (or rank-ordered) contributions of sex, empirically-derived gender facets, gender-related education, and *APOE* to healthier or relatively sustained patterns of longitudinal memory aging. Accordingly, we conducted a series of follow-up, data-driven exploratory analyses in which we applied RFA and Tree SHAP to the baseline predictors.

We describe these approaches and our analytic procedures in Supplementary Methods (Additional File 1). Briefly, we performed these analyses in two coordinated Phases. In Phase 1, we used data contributed by the entire study sample (*N* = 746) to test the relative predictive importance of sex, gender facets, and education in discriminating binary comparisons between the benchmark Low-Declining trajectory class and (a) the High-Stable trajectory class and (b) the Moderate/Normal-Declining trajectory class. In Phase 2, we used data for the genotyped subsample (*n* = 562) to test the relative predictive importance of sex, gender facets, education, and *APOE* in the two binary discrimination tasks. *APOE* was coded as an ordinal predictor representing successive increases in AD risk: 0 = ε2ε2 (least risk) and ε2ε3 (some protection); 1 = ε3ε3 (neutral risk); and 2 = ε2ε4 (some risk), ε3ε4 (more risk), and ε4ε4 (higher risk).

A complete reporting of results is provided in Additional File 1 (Supplementary Results, Supplementary Table 11, Supplementary Fig. 2). Across Phases, the random forest models (Supplementary Table 11) more reliably discriminated the extreme class comparison (Low-Declining vs. High-Stable) as compared to the neighbouring trajectory classes (Low-Declining vs. Moderate/Normal-Declining). This pattern is consistent with (a) previous applications of machine learning and data-driven applications in aging and dementia research [27, 41, 70] and (b) the marked differences in the intercept and slope parameters of the Low-Declining and High-Stable trajectory classes. Accordingly, we focus our discussion on High-Stable memory aging. See Additional File 1 for the neighbouring trajectory class comparison.

In Phase 1 (whole sample analyses), the leading predictors of High-Stable memory aging comprised four gender facets, as well as gender-related lifetime education. As shown in Supplementary Fig. 2 (Additional File 1), the most important predictor was Cognitive Activity and Brain Games, followed in order by Social and Household Management; Manual Tasks and Physical Activities; Subjective Memory Beliefs; and then education. In Phase 2 (genotyped subsample analyses), the leading predictors of High-Stable memory aging comprised five convergent and one phase-specific predictor. Regarding convergence, the same four gender facets and education predicted membership in this trajectory class. Regarding specificity, sex selectively predicted High-Stable membership when tested in the same computationally competitive context as *APOE*. As shown in Supplementary Fig. 2 (Additional File 1), Cognitive Activity and Brain Games was again the most important predictor, followed in order by Subjective Memory Beliefs; education; sex; Manual Tasks and Physical Activities; and then Social and Household Management. Across Phases, two gender facets were not amongst the leading predictive features of High-Stable membership, viz., Leisure, Socializing, and Travel; and Health Perceptions and Practices. Collectively, these results suggest that (a) multiple facets of gender are important contributors to healthier memory aging; and (b) when tested in a computationally competitive context, selected aspects of sociocultural gender and biological sex may be characterized by comparatively greater predictive importance relative to AD genetic risk. Future large-scale epidemiolocal research could formally evaluate the latter research direction by applying RFA and integrated Tree SHAP interpretations to a broader collection and biological sex and sociocultural gender variables.

## Discussion

Biological sex and sociocultural gender are interrelated constructs that mutually interact in everyday life. In research applications, an emerging perspective is that sex and gender may be represented by separate but complementary domains of characteristics and associated variables. Accordingly, a priority goal in aging and dementia research is to (a) empirically distinguish the construct domains of sex and gender and (b) test the resulting variables for separate and potentially interactive effects on longitudinal memory aging [1–5, 9, 10, 12, 23]. Such studies can contribute to clarifying patterns of results, advancing theories, and promoting precision interventions. A prominent challenge in advancing this research direction is that, although most large-scale aging and dementia datasets include at least simple binary indicators of sex/gender, few are equipped with broader and differential coverage of sex *and* gender [14, 71]. We recently reported a novel, theory-guided, and data-driven procedure for identifying, extracting, and operationalizing specific facets of aging-related lifestyle, perspectives, roles, and activities from archival aging databases that were not initially designed for explicit gender measurement [10]. In the earlier study, we applied this data-mining procedure to the VLS database and compiled a heterogeneous roster of items that corresponded to gender dimensions represented in the GOING-FWD framework [11, 17]. We then applied PCA to reduce these items into six composite gender variables that were empirically determined to represent unique (or computationally separable) facets of lifestyle gender aging. Our previous results showed that selected gender facets mediated sex differences in concurrent episodic memory performance [10].

The present data-driven study leveraged the previous cross-sectional database and extended into a 42-year longitudinal database featuring actual memory changes across an age band of 53–95 years. With this new longitudinal design, we (a) extracted data-driven memory trajectory classes and (b) examined biological sex (male, female), gender aging facets (measured as principal component variables) [10], lifetime education (a frequently investigated cognitive aging predictor with potential gender implications [3, 5, 18, 19]), and *APOE* (the most penetrant AD genetic risk factor) for separate and potentially interactive effects on memory aging (Fig. 1). Although research in this area is accumulating rapidly, we are unaware of prior research that has used such longitudinal cognitive trajectory classes as a platform for evaluating these biological and sociocultural characteristics for potential risk or protection effects.

### Identifying distinguishable classes of memory aging trajectories

We first applied data-driven LCGA to a distribution of individualized episodic memory trajectories spanning 42 years (53–95 years; RG1). Results revealed three longitudinal classes that were differentiated by intercept (level of performance) and slope (rate of decline) characteristics. The *High-Stable* class was characterized by a combination of above-average memory performance and relative maintenance over time. The *Moderate/Normal-Declining* class was characterized by a combination of mid-range memory performance and moderately steeper decline (relative to *High-Stable*). The *Low-Declining* class was characterized by a combination of the lowest level of memory performance and steeper decline. Convergent data-driven episodic memory trajectory classes have been detected in previous VLS research [27, 32, 42] and other large-scale longitudinal studies [72–74]. Observed similarities include the number of computationally discriminable classes, intercept and slope characteristics, and resulting interpretive labels. For example, one study objectively classified cognitively unimpaired older adults from the Betula Project into trajectories representing high-stable memory performance (*Maintainers*), average memory performance and decline (*Age-Typical*), and low memory performance and accelerated decline (*Decliners*) [73].

Our R3STEP prediction analyses sequentially tested sex, gender facets, and education for separate (RG2) and potentially interactive (RG3) effects on the odds of membership in the High-Stable or Moderate/Normal-Declining trajectory class as compared to the common benchmark, Low-Declining. Our focus on predicting the two highest trajectory classes responds to recent calls [5, 18, 75] for large-scale epidemiological research to integrate sex and gender in examining cognitive and brain resistance and resilience. Accordingly, our findings provide novel insight into the dynamic interplay between biological and sociocultural contributors to healthier memory aging.

### Predicting trajectory class membership

Our first set of prediction models (RG2) showed that biological sex separately predicted High-Stable membership. Specifically, females were 2.65 times more likely than males to belong to the High-Stable class. Related data-driven research has similarly reported that female sex is associated with protection for higher and sustained episodic memory performance, whereas male sex is associated with risk for comparatively lower levels and steeper decline [27, 42, 72, 73]. This pattern has been attributed to sex differences across multiple biological pathways that influence memory aging. Relevant brain-related examples include (a) atrophy in critical brain structures underlying memory function (e.g., hippocampus), whereby cognitively unimpaired males are more adversely affected than females [41, 76]; and (b) functional brain connectivity, whereby males show higher efficiency in the posterior default mode network and females show greater efficiency in frontal executive networks [77]. A notable endocrinological example is (c) circulating hormones, with males typically having higher average testosterone levels (linked to better spatial performance) and females generally having higher average estradiol levels (linked to better episodic memory performance [28]), though sex differences in estradiol become less pronounced in later life (e.g., following menopause).

A small but growing body of evidence has also attributed some sex differences in memory aging to variability in exposure to selected gender-related characteristics that may function as pathways leading toward or away from healthier memory aging [1, 3, 10, 18]. Consistent with this notion, we detected descriptive differences between males and females in three of the five gender facets that separately predicted trajectory class membership. Specifically, females were characterized by higher average component scores (i.e., greater engagement) than males in (a) Cognitive Activity and Brain Games [19] and (b) Social and Household Management [78–80]. Moreover, participation in Cognitive Activity and Brain Games separately predicted High-Stable membership, whereas participation in Social and Household Management separately predicted membership in the two highest trajectory classes. In contrast, males in our study were characterized by (a) higher average component scores (i.e., greater engagement) than females in Manual Tasks and Physical Activities [78], as well as (b) more average years of formal schooling [3, 5, 15]. Participation in Manual Tasks and Physical Activities separately predicted a lower likelihood of High-Stable (relative to Low-Declining) membership, whereas education separately predicted an increased likelihood of High-Stable membership. Interestingly, males and females were characterized by comparable average scores on two facets that separately predicted trajectory class membership, viz., Subjective Memory Beliefs and Health Perceptions and Practices. Higher scores on the former facet (i.e., more positive memory conceptions) increased the likelihood of High-Stable membership, whereas higher scores on the latter facet (i.e., poorer subjective health/greater healthcare engagement) reduced the likelihood of High-Stable membership.

Our second set of predictive models (RG3) revealed that gender facet and education effects were not moderated by sex. These results do not imply that sex and gender are independent constructs, nor that they operate independently in everyday life. Instead, they indicate that the direction and magnitude of the above-reported gender facet and education effects did not systematically differ for males relative to females. A promising set of recent studies have examined sex and gender for interactive associations with cognitive performance and decline [24, 30, 32, 39, 48, 81, 82]. However, much of this work is conducted with binary or single indirect gender variables. Accordingly, accumulating reviews [3, 5, 14–16, 18, 83] have expressed the importance of integrating longitudinal designs, evaluating both sex and gender variables, and expanding one or both from simpler binary representations to include broader complementary aspects. The present study fills in these gaps. Overall, our findings indicate that (a) the protection-enhancing or risk-reducing effects of the evaluated gender variables was robust across sex, yet (b) males and females may not equally benefit from these characteristics owing to differences in the level of engagement or exposure.

#### Cognitive activity and brain games

Findings for this gender facet revealed that higher engagement in the constituent and mentally challenging activities (e.g., jigsaw puzzles, word games) increased the likelihood of High-Stable membership. Several recent studies and reviews suggest that cognitively stimulating activities may function as a pathway towards increased brain and cognitive reserve [75, 84, 85], and such associations may translate into healthier memory aging [86]. Consistent with this notion, McFall and colleagues [27] determined—using computationally competitive RFA—that a 21-item composite measure of cognitively stimulating activities (e.g., knowledge games, reading, driving a car) was a leading predictor of membership in a data-driven trajectory class representing *Stable-Memory Aging*. Evidence from the Bronx Aging Study revealed that higher scores on a 6-item composite measure of cognitively stimulating activities (e.g., board games, playing music, group discussions) reduced risk for incident amnestic MCI [87]. Other research reported that higher scores on a 7-item composite measure of cognitively stimulating activities (e.g., playing games, museum visits, listening to radio) predicted slower memory decline and reduced AD risk [88]. We extended this prior work in two ways. First, we evaluated a homogeneous and empirically-determined Cognitive Activity and Brain Games gender variable for separate and potentially interactive associations with sex. Second, we demonstrated that, despite sex differences in patterns of engagement, such activities equally contribute to higher and sustained memory performance in aging males and females. Future research can determine whether interventions comprised of cognitively stimulating games or activities may not only enrich cognitive functioning, but also reduce or attenuate risk for impairment and dementia [89].

#### Social and household management

Modern lifestyles of many cognitively unimpaired older adults may involve engagement in management and planning of social, family, and household activities. We showed that (a) greater participation in these everyday executive-like tasks (e.g., meal planning) separately predicted an increased likelihood of belonging to the highest and moderate trajectory classes and (b) such associations were not moderated by sex. A small number of studies have evaluated related gender variables as differential predictors of cognitive [82, 90, 91] and brain aging trajectories in cognitively unimpaired cohorts [92]. Hassing and colleagues [39] evaluated the association between midlife domestic activity (as measured by a two-item composite variable) and episodic memory trajectories in later-life. Sex differences in domestic activity-memory associations were observed—increased participation predicted accelerated memory decline for females and was unassociated with memory decline for males. A more recent study reported that the total number of hours older adults engaged in six domestic activities (e.g., meal preparation, housework) did not predict concurrent memory performance, but was positively associated with gray matter, hippocampal, and frontal lobe volumes (covarying for age, sex, and education) [92]. The present study used a comprehensive set of data-driven analyses (including formal moderation tests) to show that social and managerial household tasks positively influence memory trajectories in cognitively unimpaired aging. This association may be explained in part by the cognitive demands of some executive-like activities (e.g., decision-making, planning, monitoring progress [78]) or social-planning engagement [75] accompanying these everyday gendered pursuits, which could lead to increased neurocognitive reserve [92, 93]. Educating older adults (perhaps especially males) on the potential cognitive benefits of even routine planning activities may spur higher rates of participation and in turn promote healthier memory aging.

#### Subjective memory beliefs

More positive subjective perceptions of day-to-day memory performance (e.g., ability to remember conversations, memory efficiency, perceived control) increased the likelihood of High-Stable membership. Accumulating literature has expressed that subjective cognitive decline/impairment is associated with amyloid beta deposition [94, 95], altered memory-related regional brain activity [96], as well as future objective cognitive decline and clinical transitions into MCI or AD [97, 98]. Similarly, subjective memory decline—a domain-specific construct that falls under the broader umbrella of subjective cognitive decline/impairment—not only predicts lower episodic memory performance and steeper decline [42, 48], but also increased risk for MCI and dementia [99, 100]. We advanced this line of investigation by testing (a) the predictive performance of an empirically-derived variable representing gender differences in baseline Subjective Memory Beliefs, (b) an outcome of objectively classified membership in data-driven classes representing veridical memory changes across a broad band of aging, and (c) sex differences in patterns of association. Evidence on sex moderation of subjective-objective memory associations is mixed [3, 35, 101]. Potentiated effects amongst females, males, and equivalency have all been reported. Our findings support the latter observation, in that baseline Subjective Memory Beliefs similarly predicted healthier memory aging for both males and females. Older adults characterized by higher memory self-efficacy and perceived control may be more inclined to use adaptive memory strategies [102] and to show practice-related gains on cognitive tasks [103], each of which positively contributes to memory performance [104].

#### Health perceptions and practices

Higher scores on this gender facet reflect less positive beliefs regarding one’s own health (physical, emotional), as well as increased health-seeking behaviors (e.g., hospital visits). Such unfavorable characteristics were associated with a decreased likelihood of belonging to the High-Stable (relative to Low-Declining) trajectory class. This pattern indicates that the combination of poorer subjective health and greater healthcare engagement may lead to poorer memory aging. Selected items represented in this composite variable are established risk or protection factors for accelerated memory decline [27, 32, 72, 105], MCI, and dementia [3, 18]. For example, McDermott and colleagues [27] applied RFA to a comprehensive set of 22 multi-modal variables (e.g., functional biomarkers, lifestyle characteristics) in order to determine relative importance in predicting memory resilience to AD genetic risk. Better subjective health was a top predictor of resilience for females, and lower self-reported depressive symptomatology was a leading predictor for males. More recently, Drouin et al. [41] applied LCGA to multi-wave hippocampal imaging data from the Alzheimer’s Disease Neuroimaging Initiative. A subsequent RFA showed that greater self-reported depressive symptomatology predicted membership in the lowest (most atrophied) volumetric trajectory class. Extensive literature has also linked objective physical health markers [69, 106] or multi-morbidity (e.g., frailty [44, 107, 108]) with poorer cognitive and brain aging. We extended this work by demonstrating that (a) gendered subjective health perceptions and related behaviors contributed to differential memory aging and (b) the direction and magnitude of such associations was not moderated by sex. Future research could clarify the mechanistic pathways through which varying health perceptions and practices affect memory performance and accelerated decline [75, 109].

#### Manual tasks and physical activities

We found that (a) self-reports of greater engagement in various manual household tasks (e.g., occasional household repairs) and physical activities (e.g., woodworking) were associated with a decreased likelihood of belonging to the High-Stable class and (b) such associations were not moderated by sex. Collectively, these results indicate that more frequent participation in such pursuits is associated with Low-Declining memory in cognitively unimpaired aging. Additionally, this pattern suggests a possible inverse association between the present facet and Social and Household Management—perhaps as a result of underlying differences in the extent to which the constituent activities recruit cognitive or physical capabilities. Previous research has expressed that social and household managerial tasks are more cognitively taxing than manual or physical household tasks [78], which in turn may lead to systematic differences in the extent to which these facets promote neurocognitive reserve or resilience. Consistent with this notion, McFall and colleagues [27] reported that cognitively stimulating activity is a comparatively more important contributor to longitudinal memory aging relative to physical activity. A small number of studies have examined a related gender item, occupational attainment, as a potentially modifiable risk factor for AD and related dementias [110–112]. One large-scale epidemiological study found that being a craftsman or shopkeeper doubled AD risk for males and reduced risk by 50% for females [111]. Subsequent evidence from the Kungsholmen Project indicated that manual labour positions increased AD and all-cause dementia risk, and such associations were stronger for females than males [110]. Future epidemiological studies could explore whether participation in traditionally male-typed activities (e.g., manual tasks) may have beneficial effects on non-episodic memory domains [28].

#### Lifetime education as a gendered variable

A growing number of reviews [3, 5, 18, 19, 75] have characterized education, measured as years of schooling, as a risk or protection factor for poorer memory performance, MCI, and dementia. In fact, each of the *Lancet* Commission reports on dementia has identified early-life education as amongst one of the leading modifiable factors that could be targeted for prevention or delay [106, 113, 114]. The literature on education as it relates to differential *changes* in memory performance among cognitively unimpaired aging adults is less clear [75, 115]. In the present study, we showed that education predicted healthier memory aging patterns, as each one-year increment in formal schooling increased the likelihood of High-Stable membership by 10%. Complementary findings have been reported in the small number of related works that tested education as a predictor of differential membership in data-driven memory classes [27, 32, 72, 73]. Education may exert cumulative and indirect effects on memory aging as a result of those with higher attainment (a) engaging in healthier lifestyle behaviors [74] or (b) benefitting from higher cognitive and brain reserve or resilience [5, 18, 75]. In our moderation tests, we revealed that—although females were characterized by fewer years of formal schooling—education effects did not systematically differ for males relative to females. Education has been extensively studied as a gender-related moderator of sex differences in memory performance and decline. Less research has explored whether education exerts differential effects on memory trajectories for males as compared females, and the reported findings are inconsistent [3, 5]. Taken together, our findings underscore the importance of (a) reducing gender-related disparities in access to education and (b) early intervention or prevention protocols targeting older adults with lower educational attainment. Such efforts may have positive downstream effects on memory aging for both males and females.

#### Conditional direct effect of sex and gender

Our second set of predictive models (RG3) included post-hoc tests of the conditional direct effect of sex and each successive gender variable (i.e., relative contributions when evaluated within the same multivariable model). When tested concurrently, sex (female) and the following gender facets each remained significant predictors of High-Stable memory aging: Cognitive Activity and Brain Games (increased participation), Subjective Memory Beliefs (more positive conceptions), and Health Perceptions and Practices (more positive conceptions/reduced healthcare engagement). Gender-related lifetime education (more years) also remained a significant predictor of High-Stable memory aging when sex was held constant. In contrast, sex attenuated the statistical significance (but not direction) of effects associated with two gender facets, viz., Social and Household Management and Manual Tasks and Physical Activities. Notably, these two facets were (a) the most strongly correlated with sex and (b) characterized by marked differences in average patterns of engagement. Accordingly, when tested in the same computational context, sex may account for a substantial portion of variance associated with these gendered domains, thereby attenuating the strength of their associations with longitudinal memory aging.

#### Testing *APOE* as a predictor of trajectory class membership

Recent research has emphasized the importance of evaluating whether sex and gender differences in cognitive aging are moderated by *APOE* [18]. Accordingly, our third set of R3STEP prediction models included testing *APOE* carrier status (ε4- vs. ε4+; ε2- vs. ε2+) for separate and potentially interactive effects on trajectory class membership. Findings indicated that *APOE* did not separately predict class membership, nor did it moderate sex, gender facet, and education effects. Multiple studies have reported that carrying the ε4 allele increases risk for adverse cognitive and brain aging trajectories [32, 33, 36, 38], whereas carrying the ε2 allele reduces risk [34, 51–54]. We note a prior study that examined sex, gender, and *APOE* for interactive associations with cognitive aging [37]. Participants were initially cognitively unimpaired older adults from the UK Biobank who contributed baseline data for sex (male, female) and a composite gender variable representing stereotypical feminine traits and roles. Results from cox-proportional hazard models revealed that higher gender scores (i.e., greater femininity) increased risk for incident neurocognitive impairment over 14 years of follow-up. The pattern of results detected in moderation analyses were consistent with the present study, in that gender score (and education) effects did not vary across sex and *APOE* ε4 carrier status. Collectively, these results suggest that sex and gender may operate separately from the biological pathways implicated in *APOE*-related neurodegeneration. Future research could further examine the potential dynamic interplay between sex, gender, and a broader range of AD genetic risk factors, as well as other biomedical characteristics (e.g., vascular health).

### Strengths and limitations

We acknowledge the following limitations. First, participants in the Canadian-based VLS are generally well-educated and predominantly non-Hispanic White. The present findings are therefore not known to generalize to other ethnicities, cultures, or communities. Future large-scale epidemiological datasets with diverse educational and racial/ethnic representation could examine how sex, gender, and other lived identities intersect to shape brain and cognitive aging trajectories. Second, we operationalized sex using the standard intake form from the VLS database. Consistent with previous research [4, 10, 24, 34, 37, 47, 48], participants were asked to self-report as either male or female. We acknowledge that this binary variable does not explicitly measure biological or physiological attributes that differentiate male, female, and intersex persons. Future studies are encouraged to evaluate concordant questions using a broader scope of sex-related indicators (e.g., hormones, genetics). Third, our prediction analyses tested baseline data for lifestyle gender facets and lifetime education. Other research could explore whether and how these characteristics change over time [37, 116, 117] and determine the extent that these changes are related to level and slope changes in memory performance across many years of aging.

We note the following study strengths. First, we used a well-characterized cohort of older adults that included substantial numbers of both males and females who were relatively healthy and free of neurodegenerative disease. Participants were tested on multiple occasions, which enabled us to assemble individualized memory trajectories representing a 42-year band of aging. Second, we examined our research goals using a comprehensive and complementary set of data-driven analytics. Specifically, we used LCGA to objectively discriminate classes that not only represent distinct subtypes of memory aging, but also secondary phenotypes that may be characterized by differential risk for future impairment or dementia. We integrated precision predictors directly into the LCGA using the recommended R3STEP approach. This method adjusts for potential misclassification errors in trajectory class assignments, thus producing more reliable estimates of prediction effects relative to alternative approaches (e.g., “classify-analyze” [64]). Our multi-item gender facets were empirically-determined in previous research using data-driven PCA [10]. Accordingly, these variables (a) were statistically verified as relatively homogeneous and orthogonal predictors that capture a unique facet of lifestyle gender aging and (b) required fewer statistical comparisons than would the constituent items. Third, we formally tested sex and *APOE*—the most penetrant risk factor for sporadic AD—as potential moderators using interaction terms and subsequently confirmed that the observed patterns generalized across complementary prediction modeling approaches. A limitation of our *APOE*-related analyses is that genetic data were not available for a subset of participants (*n* = 184).

### Perspectives and significance

The present study underscores the feasibility and significance of large-scale epidemiological research to integrate and evaluate sex and gender variables in aging and dementia research. Collectively, our results (a) provide empirical support for our theory-guided and data-driven gender-mining protocol [10] and (b) indicate that the evaluated predictors exerted statistically separable, non-interactive, and conditional direct effects on a 42-year span of cognitively unimpaired memory aging (53–95 years). Follow-up studies are encouraged to conceptually and operationally distinguish sex and gender and examine these constructs as separate or potentially interactive predictors of differential brain and cognitive aging trajectories. A schematic representation of a possible investigational flow is provided in Fig. 1. Results from our follow-up, data-driven exploratory analyses revealed that future research would profitably be directed towards testing the relative importance of biological sex (e.g., hormones, genetics) and gender facets (e.g., lifestyle, perspectives, roles, activities) in predicting healthier or relatively sustained patterns of memory performance and change. Such analyses could include computationally competitive machine learning analyses, viz., RFA and integrated Tree SHAP interpretations [68, 69].

## Conclusions

Our integrative data-driven analyses show that both sex *and* gender contribute to differential memory trajectory patterns in cognitively unimpaired older adults. Regarding sex, females were more likely to demonstrate High-Stable memory, whereas males were more likely to demonstrate Low-Declining memory. Regarding gender, in addition to lifetime education (higher), the following facets separately predicted High-Stable memory aging: Cognitive Activity and Brain Games (higher participation), Social and Household Management (higher participation), Subjective Memory Beliefs (higher values, which reflect more positive conceptions), Health Perceptions and Practices (lower values, which reflect more positive perceptions and lower healthcare engagement), and Manual Tasks and Physical Activities (lower participation). Moderate/Normal-Declining memory was selectively predicted by Social and Household Management activities (higher participation). Our finding that a larger number of variables predicted the highest trajectory class is consistent with (a) previous data-driven brain and cognitive aging research [27, 41, 70] and (b) the marked differences in the intercept and slope parameters of the High-Stable and benchmark Low-Declining class. An implication for precision intervention or prevention protocols is that a wide array of potentially modifiable gender facets could be targeted for the promotion of healthier memory aging. Our moderation analyses addressed an important knowledge gap by revealing that gender-related risk and protection effects generalize across such precision characteristics as sex and *APOE*.

## Supplementary Information

Supplementary Material 1

## Data Availability

The data, analytical methods, and study materials supporting the conclusions of this article are available on reasonable request. Requests to access these data and materials should be directed to Roger A. Dixon (rdixon@ualberta.ca).
