# Supplementary material for: Evaluating sex and gender as separate and interactive predictors of memory aging trajectory classes: an integrative data-driven approach
Source: Biol Sex Differ. 2026 Mar 9;17:80. doi: 10.1186/s13293-026-00864-2 (PMC13085552; doi:10.1186/s13293-026-00864-2)
Supplement: Supplementary file 1 — Supplementary Material 1 [file 13293_2026_864_MOESM1_ESM.docx]

**Supplementary Material:** Evaluating Sex and Gender as Separate and Interactive Predictors of Memory Aging Trajectory Classes: An Integrative Data-Driven Approach

**Supplementary Methods**

**Episodic Memory Measures**

For the Victoria Longitudinal Study (VLS) Word Recall [1], participants were presented with a categorized list of 30 English nouns and asked to remember as many words as possible. They were given a 2-min period to study the words and then had a 5-min period in which they wrote down as many words as they could remember. The total score of correctly recalled words served as the manifest indicator. For the Rey Auditory Verbal Learning Test [2,3], participants listened to 15 nouns and were then immediately asked to orally recall as many as possible. This procedure was repeated five times (trials A1-A5). Participants then listened to a second list (trial B1) of 15 unrelated nouns and were again asked to immediately orally recall as many as possible. Next, participants were asked to orally recall nouns from the first list (trial A6). This task provided two manifest indicators— the total number of correctly recalled nouns from Trials B1 and A6.

**Follow-Up, Data-Driven Exploratory Analyses**

We applied random forest analysis (RFA) to the baseline predictors and subsequently integrated an explainable artificial intelligence method—Tree Shapley Additive exPlanation values (Tree SHAP) [4–6]—which facilitated deep interpretation of the prediction effects generated by the black-box random forest algorithm.

**Random Forest Analysis.** Briefly, RFA is a recursive partitioning method, meaning that it combines predictions across multiple classification and regression trees, each of which is based on a random subset of participants and predictors [4–6]. This approach has several advantages relevant to the present dataset. First, it has demonstrated utility for exploring mixed-type datasets (i.e., binary and continuous predictors), as well as discriminating data-driven latent classes representing differential trajectories of brain [7] and cognitive aging [8]. Second, it can simultaneously examine a set of predictor variables for non-linear factor dependencies and complex interactions. Third, it is known to be robust to overfitting, even in studies characterized by small and/or unbalanced (uneven) subsamples. Fourth, descriptive variable importance measures that reflect the impact of each predictor on trajectory class membership can subsequently be extracted.

RFA was performed using Python 3.10.11 [9] with the RandomForestClassifier from the scikit-learn package [10]. In Phase 1 (i.e., whole sample analyses), we used the following hyperparameters for each binary discrimination task: (a) Low-Declining *vs*. High-Stable = max_depth: 3, max_features: None, n_estimators: 1000; and (b) Low-Declining *vs*. Moderate/Normal-Declining = max_depth: 3, max_features: None, n_estimators: 100. In Phase 2 (i.e., genotyped subsample analyses), we used the following hyperparameters for each binary discrimination task: (a) Low-Declining *vs*. High-Stable = max_depth: 3, max_features: None, n_estimators: 500; and (b) Low-Declining *vs*. Moderate/Normal-Declining = max_depth: 3, max_features: 'sqrt', n_estimators: 2000.

In both phases of the RFA, model performance was evaluated using stratified k-fold cross-validation [4–6]. This approach is recommended when the subsamples comprising the binary discrimination tasks are small and/or unbalanced. These models ensure that each k-fold (or subsample) contains roughly the same proportion of each “subgroup” (or latent trajectory class) as is represented in the overall sample. Specifically, we used stratified five-fold cross-validation to divide each pairwise dataset into five equally sized folds. Four of the five folds were used for training and the remaining fold was used for testing. This process was repeated five times, until each fold had been used once for testing. The model then returned the five-evaluation metrics, which were averaged across the cross-validation folds.

The first and main metric was the area under the receiver operating characteristic curve (AUC), a summary measure of the model’s ability to distinguish between the benchmark class and the High-Stable or Moderate/Normal-Declining trajectory classes. AUC is interpreted such that 0.5 represents chance, 0.5–0.69 represents poor discrimination, 0.7–0.79 represents acceptable discrimination, 0.8–0.89 represents excellent discrimination, and ≥ 0.9 represents outstanding discrimination. The second metric was accuracy, which refers to the total percentage of participants who were correctly classified as belonging to either the Low-Declining class or comparison class (i.e., High-Stable; Moderate/Normal-Declining). Accuracy is calculated as the fraction of true positives and true negatives over all model classifications. The third metric was precision, which represents the percentage of participants who were correctly classified into either the High-Stable or Moderate/Normal-Declining class (calculated as true positives / (true positives + false positives)). The fourth metric was sensitivity (or recall), which reflects the percentage of participants from the comparison class (i.e., High-Stable; Moderate/Normal-Declining) who were correctly classified as such (calculated as true positives / (true positives + false negatives)). The fifth metric was F1 score, an overall measure of model performance that represents a specific combination of precision and sensitivity. Technically, the F1 score is the “harmonic mean” of precision and sensitivity. As such, it is calculated as 2 x (precision × sensitivity) / (precision + sensitivity). Values for the latter four metrics (i.e., accuracy, precision, sensitivity, F1 score) also ranged between 0 and 1, with higher values denoting better classification performance. In studies with unbalanced subsamples, AUC and F1 score are the most robust indicators of model performance. We report these five metrics for each binary discrimination task but focus on AUC and F1 score when evaluating and interpreting (or assigning a qualitative label) to model fit.

**Tree Shapley Additive exPlanation values**. Although RFA provides a robust and computationally competitive context from which to detect leading discriminative predictors—enhanced interpretations of the prediction patterns are afforded by Tree SHAP values [4–6]. For each binary discrimination task, Tree SHAP (a) provides a unified framework for determining the relative importance (or model contribution) of the considered predictors and (b) generates a waterfall plot. The waterfall plot advances interpretation by (a) depicting the predictors in descending order of global importance and (b) providing a visual representation of results from the RFA. Specifically, this plot depicts the individual and cumulative ratio of the predictors’ contribution to the classification model (represented by the bars and curved line, respectively).

**Supplementary Results**

**Follow-Up, Data-Driven Exploratory Analyses**

**Random forest analysis: Phase 1.** Performance metrics for the random forest model evaluating the relative predictive importance of sex, gender facets, and education are reported in Supplementary Table 10 (top panel). Results indicated (a) near acceptable discrimination of the Low-Declining and High-Stable trajectory classes; and (b) comparatively more modest discrimination of the Low-Declining and Moderate/Normal-Declining trajectory classes.

Supplementary Figure 2 displays the Tree SHAP waterfall plots for binary discrimination of the Low-Declining and (a) High-Stable trajectory classes (Panel A) and (b) Moderate/Normal-Declining trajectory classes (Panel B). In Panel A, the top five predictors are bolded for four reasons. First, they have the highest global importance ratings (indexed by the composition ratio (i.e., blue bars; see top of panel for scale and Figure legend for interpretation)). Second, there is an evident elbow (or break in the distribution) in the cumulative ratio at this cut-off. Third, they collectively explained more than 80% of the model (indexed by the cumulative ratio (i.e., blue curved line; see bottom of panel for scale and Figure legend for interpretation)). Fourth, predictors below this cut-off contributed comparatively less to the classification model. The five leading predictors (in descending order of importance) are: Cognitive Activity and Brain Games; Social and Household Management; Manual Tasks and Physical Activities; Subjective Memory Beliefs; and education. In Panel B, the top six predictors are bolded based on these same considerations. The leading predictors (in descending order of importance) are: Cognitive Activity and Brain Games; Social and Household Management; Subjective Memory Beliefs; Leisure, Socializing and Travel; Health Perceptions and Practices; Manual Tasks and Physical Activities.

A comparison of the leading predictors extracted across these two binary discrimination tasks revealed both convergent (i.e., variables predicting membership in each of the higher trajectory classes) and class-specific predictors (i.e., variables predicting membership in only one of the two higher trajectory classes). Regarding convergence, four predictors were associated with membership in each of the higher trajectory classes: Cognitive Activity and Brain Games; Social and Household Management; Subjective Memory Beliefs; and Manual Tasks and Physical Activities. Regarding specificity, membership in the High-Stable class was selectively predicted by education, whereas Moderate/Normal-Declining membership was selectively predicted by Leisure, Socializing, and Travel, as well as Health Perceptions and Practices.

**Random forest analysis: Phase 2.** Supplementary Table 10 (lower panel) displays performance metrics for the random forest model evaluating the relative predictive importance of sex, gender facets, education, and *APOE*. Results indicated (a) near acceptable discrimination of the Low-Declining and High-Stable trajectory classes; and (b) comparatively more modest discrimination of the Low-Declining and Moderate/Normal-Declining trajectory classes.

In Supplementary Figure 2, we display the Tree SHAP waterfall plots for binary discrimination of the Low-Declining and (a) High-Stable trajectory classes (Panel C) and (b) Moderate/Normal-Declining trajectory classes (Panel D). The leading predictors of the High-Stable class (in descending order of importance) are: Cognitive Activity and Brain Games; Subjective Memory Beliefs; education; sex; Manual Tasks and Physical Activities; and Social and Household Management. The leading predictors of the Moderate/Normal-Declining class (in descending order of importance) are: Subjective Memory Beliefs; Social and Household Management; Cognitive Activity and Brain Games; Manual Tasks and Physical Activities; and Leisure, Socializing, and Travel.

A comparison of the leading predictors extracted across these two binary discrimination tasks revealed both convergent and class-specific predictors. Regarding convergence, four predictors were associated with membership in each of the higher trajectory classes: Cognitive Activity and Brain Games; Social and Household Management; Subjective Memory Beliefs; and Manual Tasks and Physical Activities. Regarding specificity, membership in the High-Stable class was selectively predicted by education and sex, whereas membership in the Moderate/Normal-Declining class was selectively predicted by Leisure, Socializing, and Travel.

**Supplementary Table 1.** Principal Component Loadings of the Final Set of Gender Facet Items (*N* = 37)

|  |  | **Gender Facet (Principal Component)** | | | | | |
| --- | --- | --- | --- | --- | --- | --- | --- |
| **Item** | | **1** | **2** | **3** | **4** | **5** | **6** |
| Drive a car | | **0.40** | 0.23 | 0.23 | -0.14 | -0.01 | 0.10 |
| Do household repairs | | **0.86** | -0.08 | 0.07 | 0.04 | -0.08 | -0.01 |
| Repair car, lawnmower, or other mechanical device | | **0.83** | -0.20 | -0.01 | 0.04 | -0.04 | -0.03 |
| Purchase item requiring set-up or assembly | | **0.69** | -0.08 | 0.12 | 0.11 | 0.00 | 0.02 |
| Woodworking, carpentry, or furniture refinishing | | **0.78** | -0.13 | -0.02 | 0.04 | -0.03 | -0.05 |
| Physical activity derived scale | | **0.38** | 0.25 | 0.19 | 0.05 | -0.24 | -.16 |
| Meal preparation | | -0.04 | **0.73** | -0.05 | 0.05 | -0.04 | 0.01 |
| Housework | | -0.05 | **0.68** | 0.02 | 0.03 | -0.07 | 0.00 |
| Food shopping | | 0.01 | **0.68** | 0.03 | 0.00 | 0.03 | -0.04 |
| Sewing or knitting | | -0.28 | **0.44** | 0.03 | 0.22 | 0.04 | 0.04 |
| Visiting relatives, friends, or neighbours | | -0.04 | **0.36** | 0.27 | 0.17 | 0.05 | 0.08 |
| External memory strategy derived scale | | -0.16 | **0.32** | 0.13 | -0.16 | 0.02 | -0.21 |
| Writing letters | | 0.08 | 0.19 | **0.41** | 0.01 | 0.03 | -0.78 |
| Travel within province | | 0.07 | 0.00 | **0.74** | 0.08 | -0.07 | -0.01 |
| Travel outside of province (but within Canada) | | 0.04 | -0.10 | **0.73** | 0.09 | -0.12 | 0.02 |
| Travel in foreign country | | 0.11 | 0.01 | **0.66** | 0.02 | -0.08 | 0.09 |
| Give dinner party or party for friends | | 0.14 | 0.29 | **0.51** | 0.15 | -0.08 | 0.03 |
| Eat out at restaurants | | -.06 | -0.02 | **0.36** | -0.02 | 0.12 | 0.07 |
| Crossword puzzles, acrostics, or anagrams | | -0.12 | 0.02 | -0.11 | **0.55** | -0.04 | -0.10 |
| Jigsaw puzzles | | 0.05 | 0.17 | 0.00 | **0.50** | 0.03 | 0.00 |
| Boards games such as chess and checkers | | 0.15 | 0.01 | 0.10 | **0.60** | 0.04 | 0.03 |
| Knowledge games such as Trivial pursuit | | 0.08 | -0.02 | 0.16 | **0.65** | 0.08 | 0.13 |
| Word games such as Scrabble | | -0.03 | 0.01 | 0.11 | **0.73** | -0.03 | 0.03 |
| Go to the hospital in past 4 weeks | | 0.02 | 0.00 | 0.05 | 0.01 | **0.37** | 0.06 |
| Seen a doctor in past 4 weeks | | 0.00 | 0.04 | 0.07 | -0.01 | **0.62** | 0.03 |
| Number of times seeing a doctor in the past year | | -0.07 | 0.01 | -0.01 | 0.00 | **0.60** | 0.01 |
| Overall health relative to peers | | -0.08 | -0.08 | -0.06 | 0.2 | **0.75** | -0.06 |
| Overall health relative to perfect | | -0.07 | -0.12 | -0.09 | 0.04 | **0.79** | -0.11 |
| Depression derived scale | | -0.01 | 0.12 | -0.22 | 0.04 | **0.36** | -0.13 |
| As good at remembering as I ever was | | 0.02 | -.02 | 0.07 | -0.33 | 0.28 | **0.88** |
| Remember things as well as always | | 0.01 | -0.06 | 0.04 | -0.04 | -0.01 | **0.85** |
| Good at remembering conversations | | -0.08 | 0.09 | 0.10 | 0.05 | -0.02 | **0.45** |
| Memory anxiety derived scale | | -0.07 | 0.15 | -0.01 | -0.07 | 0.17 | **-0.48** |
| Little control over my memory ability | | 0.05 | 0.17 | 0.08 | 0.04 | -0.09 | **0.39** |
| If I keep using my memory I will never lose it | | 0.03 | -0.13 | -0.03 | 0.03 | 0.09 | **0.32** |
| Less efficient at remembering things now | | 0.03 | 0.00 | 0.04 | -0.09 | 0.02 | **0.83** |
| Memory recruitment derived scale | | 0.10 | -0.21 | 0.20 | -0.03 | 0.00 | **-0.36** |
| **Variance explained (%)** | | 8.4% | 6.1% | 7.6% | 4.6% | 4.7% | 10.5% |

*Note*. Component loadings with an absolute value greater than 0.30 are bolded. Component 1: Manual Tasks and Physical Activities; Component 2: Social and Household Management; Component 3: Leisure, Socializing, and Travel; Component 4: Cognitive Activity and Brain Games; Component 5: Heath Perceptions and Practices; Component 6: Subjective Memory Beliefs.

| **Supplementary Table 2.** Correlation Coefficient Between Sex, Six Gender Facets, and Education | | | | | | | | |
| --- | --- | --- | --- | --- | --- | --- | --- | --- |
|  | Sex | Manual Tasks and Physical Activities | Social and Household Management | Leisure, Socializing, and Travel | Cognitive Activity and Brain Games | Health Perceptions and Practices | Subjective Memory Beliefs | Education |
| Sex | – | -0.55^**^ | 0.51^**^ | -0.02 | 0.11^**^ | 0.06 | 0.07 | -0.15^**^ |
| Manual Tasks and Physical Activities |  | – | .00 | .00 | .00 | .00 | .00 | .20^**^ |
| Social and Household Management |  |  | – | .00 | .00 | .00 | .00 | -.05 |
| Leisure, Socializing, and Travel |  |  |  | – | .00 | .00 | .00 | .18^**^ |
| Cognitive Activity and Brain Games |  |  |  |  | – | .00 | .00 | 0.02 |
| Heath Perceptions and Practices |  |  |  |  |  | – | .00 | -.03 |
| Subjective Memory Beliefs |  |  |  |  |  |  | – | .05 |
| Education |  |  |  |  |  |  |  | – |

***Note.*** Sex is coded as 0 = male, 1 = female. Coefficients represent point-biserial correlations and Pearson’s correlation (as appropriate). Collectively, these results (a) indicate that the gender facet variables are orthogonal (or uncorrelated) predictors that capture a unique aspect of gender aging; (b) demonstrate significant heterogeneity in patterns of association between the baseline predictors; and (c) provide clear statistical justification for testing and reporting the separate effect of sex, gender facets, and education on trajectory class membership.

^**^ *p* < .01 ^*^ *p* < .05

**Supplementary Table 3.** Fit Indices for Confirmatory Factor Analysis and Measurement Invariance Testing of the Latent Memory Variable

| **Model** | **﻿χ^2^** | ***df*** | ***p*** | **RMSEA** | **CFI** | **SRMR** | **﻿ ﻿ΔCFI** |
| --- | --- | --- | --- | --- | --- | --- | --- |
| Configural invariance | 17.95 | 12 | 0.11 | 0.03 [0, 0.05] | 1.0 | 0.01 | -- |
| Metric invariance | 42.94 | 16 | < .001 | 0.05 [0.03, 0.07] | 0.99 | 0.06 | <.01 |
| **Scalar invariance** | **58.18** | **18** | **<.001** | **0.06 [0.04, 0.07]** | **0.98** | **0.06** | **<.01** |

*Note.* χ2, chi-square test of model fit; *df*, degrees of freedom for model fit; RMSEA, root mean square error of approximation; RMSEA is shown with 90% confidence intervals; CFI, comparative fit index; SRMR, standardized root mean square residual; ﻿ΔCFI = change in CFI. The best fitting model is bolded.

**Supplementary Table 4**. Fit Indices for Unconditional Latent Growth Models of Memory

| **Model** | **(-)2*LL*** | **npar free** | **﻿AIC** | **BIC** | ***D*** | **﻿ ﻿Δ*df*** |
| --- | --- | --- | --- | --- | --- | --- |
| Fixed intercept | 4,731.91 | 4 | 4,739.92 | 4,758.37 | -- | -- |
| Random intercept | 3,524.82 | 5 | 3,534.82 | 3,557.89 | 1,207.09^***^ | 1 |
| Random intercept, fixed slope | 2,337.58 | 6 | 2,349.58 | 2,377.26 | 1,187.24^***^ | 1 |
| **Random intercept, random slope** | **2,168.06** | **8** | **2,184.06** | **2,220.98** | **169.52**^**^ | **2** |

*Note.* -2*LL*, -2 log-likelihood; npar, number of parameters; AIC, Akaike information criterion; BIC, Bayesian information criterion; *D*, difference statistic. The best fitting model is bolded.

^***^ *p* < .001

**Supplementary Table 5.** Interaction Terms from R3STEP Prediction Models Evaluating Sex as a Potential Moderator of Gender Facet and Education Effects on Trajectory Class Membership

|  | **Trajectory Class** | | | | |
| --- | --- | --- | --- | --- | --- |
|  | | *High-Stable* | | *Moderate/Normal-Declining* | |
|  | | OR [95% CI] | | OR [95% CI] | |
| ***Model 1*** | | |  | |  |
| Sex × Manual Tasks and Physical Activities | | | 1.09 [0.62, 1.93] | | 1.13 [0.66, 1.94] |
| ***Model 2*** | | |  | |  |
| Sex × Social and Household Management | | | 1.10 [0.66, 1.83] | | 0.86 [0.51, 1.42] |
| ***Model 3*** | | |  | |  |
| Sex × Leisure, Socializing, and Travel | | | 0.88 [0.54, 1.45] | | 0.83 [0.51, 1.34] |
| ***Model 4*** | | |  | |  |
| Sex × Cognitive Activity and Brain Games | | | 0.92 [0.54, 1.57] | | 1.15 [0.68, 1.94] |
| ***Model 5*** | | |  | |  |
| Sex × Health Perceptions and Practices | | | 0.86 [0.52, 1.43] | | 1.32 [0.81, 2.15] |
| ***Model 6*** | | |  | |  |
| Sex × Subjective Memory Beliefs | | | 0.99 [0.63, 1.57] | | 1.16 [0.73, 1.82] |
| ***Model 7*** | | |  | |  |
| Sex × Education (years) | | | 0.97 [0.82, 1.15] | | 0.88 [0.75, 1.04] |

*Note*. OR, odds ratio; CI, confidence interval. Sex was categorized as 0 = male, 1 = female. For each model: (a) the *Low-Declining* class was specified as the common benchmark and (b) two main effects (results not shown) and one interaction term was included.

**Supplementary Table 6.** Interaction Terms from “Classify-Analyze” Prediction Models Evaluating Sex as a Potential Moderator of Gender Facet and Education Effects on Trajectory Class Membership

|  | **Trajectory Class** | | | | |
| --- | --- | --- | --- | --- | --- |
|  | | *High-Stable* | | *Moderate/Normal-Declining* | |
|  | | OR [95% CI] | | OR [95% CI] | |
| ***Model 1*** | | |  | |  |
| Sex × Manual Tasks and Physical Activities | | | 1.09 [0.64, 1.84] | | 1.12 [0.69, 1.78] |
| ***Model 2*** | | |  | |  |
| Sex × Social and Household Management | | | 1.09 [0.68, 1.75] | | 0.88 [0.57, 1.36] |
| ***Model 3*** | | |  | |  |
| Sex × Leisure, Socializing, and Travel | | | 0.90 [0.57, 1.41] | | 0.85 [0.56, 1.28] |
| ***Model 4*** | | |  | |  |
| Sex × Cognitive Activity and Brain Games | | | 0.93 [0.57, 1.50] | | 1.12 [0,72, 1.74] |
| ***Model 5*** | | |  | |  |
| Sex × Health Perceptions and Practices | | | 0.86 [0.54, 1.39] | | 1.25 [0.82, 1.90] |
| ***Model 6*** | | |  | |  |
| Sex × Subjective Memory Beliefs | | | 1.0 [0.65, 1.52] | | 1.14 [0.77, 1.69] |
| ***Model 7*** | | |  | |  |
| Sex × Education (years) | | | 0.98 [0.84, 1.15] | | 0.90 [0.78, 1.04] |

*Note*. OR, odds ratio; CI, confidence interval. Sex was categorized as 0 = male, 1 = female.

For each model: (a) the *Low-Declining* class was specified as the common benchmark and (b) two main effects (results not shown) and one interaction term was included.

**Supplementary Table 7.** Interaction Terms from R3STEP Prediction Models Evaluating *APOE* ε4 Carrier Status as a Potential Moderator of Sex, Gender Facet, and Education Effects on Trajectory Class Membership

|  | **Trajectory Class** | | | | | | | |
| --- | --- | --- | --- | --- | --- | --- | --- | --- |
|  | | *High-Stable* | | | | *Moderate/Normal-Declining* | | |
|  | | OR [95% CI] | | | | OR [95% CI] | | |
| ***Model 1*** | | |  | | | |  | |
| *APOE* ε4 × Sex | | | 0.49 [0.13, 1.84] | | | | 0.61 [0.18, 2.13] | |
| ***Model 2*** | | | | |  | | |  |
| *APOE* ε4 × Manual Tasks and Physical Activities | | | | 1.03 [0.54, 1.97] | | | | 1.25 [0.72, 2.18] |
| ***Model 3*** | | |  | | | |  | |
| *APOE* ε4 × Social and Household Management | | | 0.82 [0.40, 1.69] | | | | 1.03 [0.54, 1.96] | |
| ***Model 4*** | | |  | | | |  | |
| *APOE* ε4 × Leisure, Socializing, and Travel | | | 1.19 [0.61, 2.30] | | | | 0.94 [0.48, 1.83] | |
| ***Model 5*** | | |  | | | |  | |
| *APOE* ε4 × Cognitive Activity and Brain Games | | | 0.90 [0.46, 1.77] | | | | 0.75 [0.38, 1.50] | |
| ***Model 6*** | | |  | | | |  | |
| *APOE* ε4 × Health Perceptions and Practices | | | 1.51 [0.76, 3.0] | | | | 1.49 [0.75, 2.99] | |
| ***Model 7*** | | |  | | | |  | |
| *APOE* ε4 × Subjective Memory Beliefs | | | 1.0 [0.57, 1.78] | | | | 1.70 [0.95, 3.03] | |
| ***Model 8*** | | |  | | | |  | |
| *APOE* ε4 × Education (years) | | | 1.00 [0.82, 1.23] | | | | 0.92 [0.75, 1.13] | |

*Note*. OR, odds ratio; CI, confidence interval; *APOE*, Apolipoprotein E. *APOE* ε4 carrier status was categorized as 0 = non-carrier, 1 = carrier. Sex was categorized as 0 = male, 1 = female.

For each model: (a) the *Low-Declining* class was specified as the common benchmark and (b) two main effects (results not shown) and one interaction term was included. Results are based on the subset of genotyped participants (*n* = 536).

**Supplementary Table 8.** Interaction Terms from R3STEP Models Evaluating *APOE* ε2 Carrier Status as a Potential Moderator of Sex, Gender Facet, and Education Effects on Trajectory Class

Membership

|  | **Trajectory Class** | | | | | | | |
| --- | --- | --- | --- | --- | --- | --- | --- | --- |
|  | | *High-Stable* | | | *Moderate/Normal-Declining* | | | |
|  | | OR [95% CI] | | | OR [95% CI] | | | |
| ***Model 1*** | | |  | | |  | | |
| *APOE* ε2 × Sex | | | 3.13 [0.32, 30.83] | | | 1.72 [0.20, 14.89] | | |
| ***Model 2*** | | | |  | | |  | |
| *APOE* ε2 × Manual Tasks and Physical Activities | | | | 0.84 [0.32, 2.23] | | | | 1.00 [0.42, 2.37] |
| ***Model 3*** | | |  | | |  | | |
| *APOE* ε2 × Social and Household Management | | | 1.76 [0.68, 4.52] | | | 1.60 [0.69, 3.73] | | |
| ***Model 4*** | | |  | | |  | | |
| *APOE* ε2 × Leisure, Socializing, and Travel | | | 0.81 [0.33, 1.97] | | | 0.63 [0.25, 1.57] | | |
| ***Model 5*** | | |  | | |  | | |
| *APOE* ε2 × Cognitive Activity and Brain Games | | | 1.83 [0.56, 5.99] | | | 1.20 [0.34, 4.20] | | |
| ***Model 6*** | | |  | | |  | | |
| *APOE* ε2 × Health Perceptions and Practices | | | 1.20 [0.29, 4.98] | | | 1.01 [0.23, 4.44] | | |
| ***Model 7*** | | |  | | |  | | |
| *APOE* ε2 × Subjective Memory Beliefs | | | 1.01 [0.45, 2.25] | | | 0.60 [0.28, 1.31] | | |
| ***Model 8*** | | |  | | |  | | |
| *APOE* ε2 × Education (years) | | | 0.96 [0.67, 1.37] | | | 0.97 [0.66, 1.41] | | |

*Note*. OR, odds ratio; CI, confidence interval; *APOE,* Apolipoprotein E. *APOE* ε2 carrier status was categorized as 0 = non-carrier, 1 = carrier. Sex was categorized as 0 = male, 1 = female.

For each model: (a) the *Low-Declining* class was specified as the common benchmark and (b) two main effects (results not shown) and one interaction term was included. Results are based on the subset of genotyped participants (*n* = 536).

**Supplementary Table 9.** Results from R3STEP Prediction Models Evaluating Conditional Direct Effects of *APOE* ε4 Carrier Status*,* Sex, Gender Facets, and Education on Trajectory Class Membership

|  | **Trajectory Class** | | | | | | | |
| --- | --- | --- | --- | --- | --- | --- | --- | --- |
|  | | *High-Stable* | | | | *Moderate/Normal-Declining* | | |
|  | | OR [95% CI] | | | | OR [95% CI] | | |
| ***Model 1*** | | |  | | | |  | |
| *APOE* ε4 | | | 0.62 [0.33, 1.17] | | | | 0.81 [0.43, 1.49] | |
| Sex | | | 3.41 [1.91 6.09] ^*^ | | | | 1.78 [1.02, 3.01] ^*^ | |
| ***Model 2*** | | | | |  | | |  |
| *APOE* ε4 | | | | 0.66 [0.36 1.24] | | | | 0.82 [0.45, 1.53] |
| Manual Tasks and Physical Activities | | | | 0.71 [0.54, 0.93] ^*^ | | | | 0.87 [0.68, 1.10] |
| ***Model 3*** | | |  | | | |  | |
| *APOE* ε4 | | | 0.58 [0.31, 1.08] | | | | 0.77 [0.42, 1.42] | |
| Social and Household Management | | | 1.54 [1.15, 2.01] ^*^ | | | | 1.29 [0.98, 1.70] | |
| ***Model 4*** | | |  | | | |  | |
| *APOE* ε4 | | | 0.64 [0.34, 1.18] | | | | 0.81 [0.44, 1.49] | |
| Leisure, Socializing, and Travel | | | 1.26 [0.95, 1.68] | | | | 1.17 [0.87, 1.57] | |
| ***Model 5*** | | |  | | | |  | |
| *APOE* ε4 | | | 0.58 [0.31, 1.08] | | | | 0.79 [0.43, 1.45] | |
| Cognitive Activity and Brain Games | | | 1.73 [1.28, 2.34] ^*^ | | | | 1.22 [0.89, 1.67] | |
| ***Model 6*** | | |  | | | |  | |
| *APOE* ε4 | | | 0.60 [0.32, 1.11] | | | | 0.81 [0.44, 1.49] | |
| Health Perceptions and Practices | | | 0.81 [0.62, 1.07] | | | | 1.06 [0.81, 1.39] | |
| ***Model 7*** | | |  | | | |  | |
| *APOE* ε4 | | | 0.62 [0.33, 1.15] | | | | 0.79 [0.43, 1.46] | |
| Subjective Memory Beliefs | | | 1.45 [1.10, 1.91] ^*^ | | | | 1.25 [0.96, 1.64] | |
| ***Model 8*** | | |  | | | |  | |
| *APOE* ε4 | | | 0.59 [0.32, 1.10] | | | | 0.80 [0.44, 1.48] | |
| Education (years) | | | 1.14 [1.04, 1.25] ^*^ | | | | 1.0 [0.91, 1.10] | |

*Note*. OR, odds ratio; CI, confidence interval; *APOE,* Apolipoprotein E. *APOE* ε4 carrier status was categorized as 0 = non-carrier, 1 = carrier. Sex was categorized as 0 = male, 1 = female. For each model, (a) the *Low-Declining* class was specified as the common benchmark and (b) two main effect terms were included. Results are based on the subset of genotyped participants (*n* = 536).

^*^ Denotes statistically significant prediction effects (based on a 95% CI that does not include 0).

**Supplementary Table 10.** Results from R3STEP Prediction Models Evaluating Conditional Direct Effects of *APOE* ε2 Carrier Status*,* Sex, Gender Facets, and Education on Trajectory Class Membership

|  | **Trajectory Class** | | | | | | | |
| --- | --- | --- | --- | --- | --- | --- | --- | --- |
|  | | *High-Stable* | | | *Moderate/Normal-Declining* | | | |
|  | | OR [95% CI] | | | OR [95% CI] | | | |
| ***Model 1*** | | |  | | |  | | |
| *APOE* ε2 | | | 2.15 [0.79, 5.82] | | | 2.28 [0.82, 6.33] | | |
| Sex | | | 3.35 [1.88, 5.98] ^*^ | | | 1.75 [1.00, 3.06] ^*^ | | |
| ***Model 2*** | | | |  | | |  | |
| *APOE* ε2 | | | | 2.22 [0.81, 6.11] | | | | 2.35 [0.84, 6.61] |
| Manual Tasks and Physical Activities | | | | 0.70 [0.53, 0.92] ^*^ | | | | 0.86 [0.68, 1.10] |
| ***Model 3*** | | |  | | |  | | |
| *APOE* ε2 | | | 2.33 [0.87, 6.23] | | | 2.39 0.88, 6.52] | | |
| Social and Household Management | | | 1.52 [1.13, 2.05] ^*^ | | | 1.29 [0.97, 1.72] | | |
| ***Model 4*** | | |  | | |  | | |
| *APOE* ε2 | | | 2.57 [0.81, 6.30] | | | 2.39 [0.84, 6.82] | | |
| Leisure, Socializing, and Travel | | | 1.27 [0.96, 1.69] | | | 1.17 [0.87, 1.58] | | |
| ***Model 5*** | | |  | | |  | | |
| *APOE* ε2 | | | 2.40 [0.88, 6.53] | | | 2.40 [0.86, 6.68] | | |
| Cognitive Activity and Brain Games | | | 1.72 [1.27, 2.34] ^*^ | | | 1.22 [0.89, 1.68] | | |
| ***Model 6*** | | |  | | |  | | |
| *APOE* ε2 | | | 2.35 [0.85, 6.50] | | | 2.35 [0.83, 6.62] | | |
| Health Perceptions and Practices | | | 0.81 [0.62, 1.07] | | | 1.05 [0.79, 1.39] | | |
| ***Model 7*** | | |  | | |  | | |
| *APOE* ε2 | | | 2.22 [0.79, 6.21] | | | 2.37 [0.83, 6.81] | | |
| Subjective Memory Beliefs | | | 1.45 [1.10, 1.90] ^*^ | | | 1.25 [0.95, 1.64] | | |
| ***Model 8*** | | |  | | |  | | |
| *APOE* ε2 | | | 2.36 [0.85, 6.58] | | | 2.37 [0.84, 6.69] | | |
| Education (years) | | | 1.13 [1.03, 1.25] ^*^ | | | 1.00 [0.91, 1.11] | | |

*Note*. OR, odds ratio; CI, confidence interval; *APOE,* Apolipoprotein E. *APOE* ε2 carrier status was categorized as 0 = non-carrier, 1 = carrier. Sex was categorized as 0 = male, 1 = female.

For each model, (a) the *Low-Declining* class was specified as the common benchmark and (b) two main effect terms were included. Results are based on the subset of genotyped participants (*n* = 536).

^*^ Denotes statistically significant prediction effects (based on a 95% CI that does not include 0).

**Supplementary Table 11.** Model Evaluation Metrics for both Phases of the Random Forest Analysis

| **Binary Discrimination Task** | **AUC** | **Accuracy** | **Precision** | **Sensitivity** | **F_1_ score** |
| --- | --- | --- | --- | --- | --- |
| **Phase 1: Relative predictive importance of sex, gender facets, and education** | | | | | |
| Low-Declining *vs.* High-Stable | 0.68 (0.04) | 0.61 (0.07) | 0.47 (0.09) | 0.55 (0.06) | 0.50 (0.07) |
| Low-Declining *vs.* Moderate/Normal-Declining | 0.56 (0.05) | 0.60 (0.03) | 0.29 (0.03) | 0.34 (0.07) | 0.31 (0.04) |
| **Phase 2: Relative predictive importance of sex, gender facets, education, and *APOE*** | | | | | |
| Low-Declining *vs.* High-Stable | 0.66 (0.07) | 0.67 (0.05) | 0.51 (0.08) | 0.47 (0.11) | 0.49 (0.09) |
| Low-Declining *vs.* Moderate/Normal-Declining | 0.51 (0.06) | 0.60 (0.06) | 0.26 (0.11) | 0.30 (0.13) | 0.27 (0.11) |

*Note.* Evaluation metrics reflect average performance of the random forest model across the five cross-validation folds. Standard deviations are reported in parentheses. Each evaluation metric ranges between 0 and 1 (higher values denote better performance; for detail see Supplementary Methods). AUC, area under the receiver operating characteristic curve; *APOE*, Apolipoprotein E. *APOE* was coded as an ordinal predictor representing successive increases in AD risk: 0 = ε2ε2 (least risk) and ε2ε3 (some protection); 1 = ε3ε3 (neutral risk); and 2 = ε2ε4 (some risk), ε3ε4 (more risk), and ε4ε4 (higher risk). Results are based on the subset of genotyped participants (*n* = 562, as carriers the *APOE* ε2ε4 allele were included).


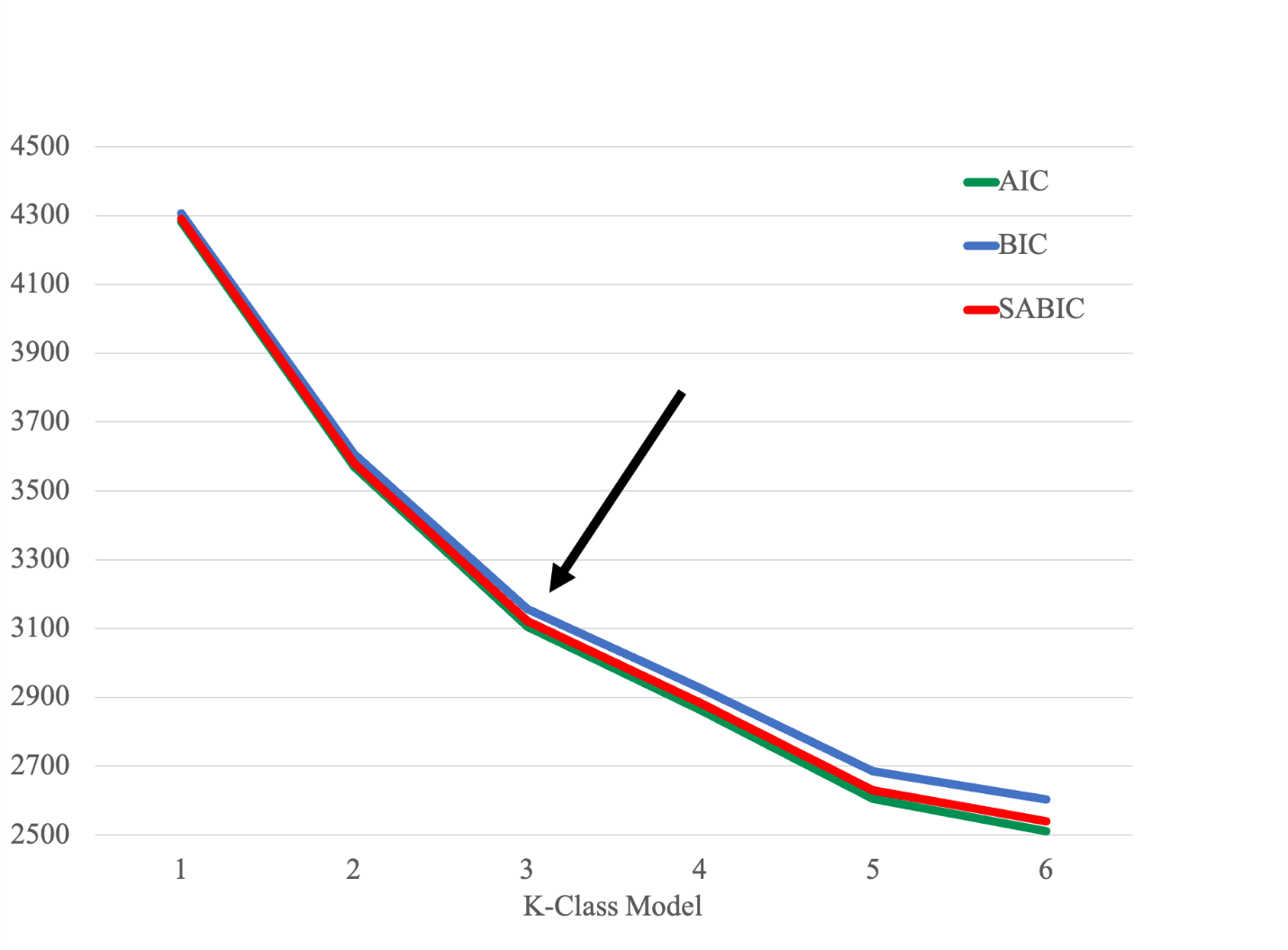


**Supplementary Figure 1.** Scree plot of Akaike Information Criterion (AIC), Bayesian Information Criterion (BIC), and sample-size adjusted BIC (SABIC) for each *k*-class model from the unconditional latent class growth analysis of memory. The arrow represents the inflection point (i.e., the “elbow” or break in the distribution of the relative fit indices). The three-class solution (*k* = 3) was selected as the best-fitting model.

**
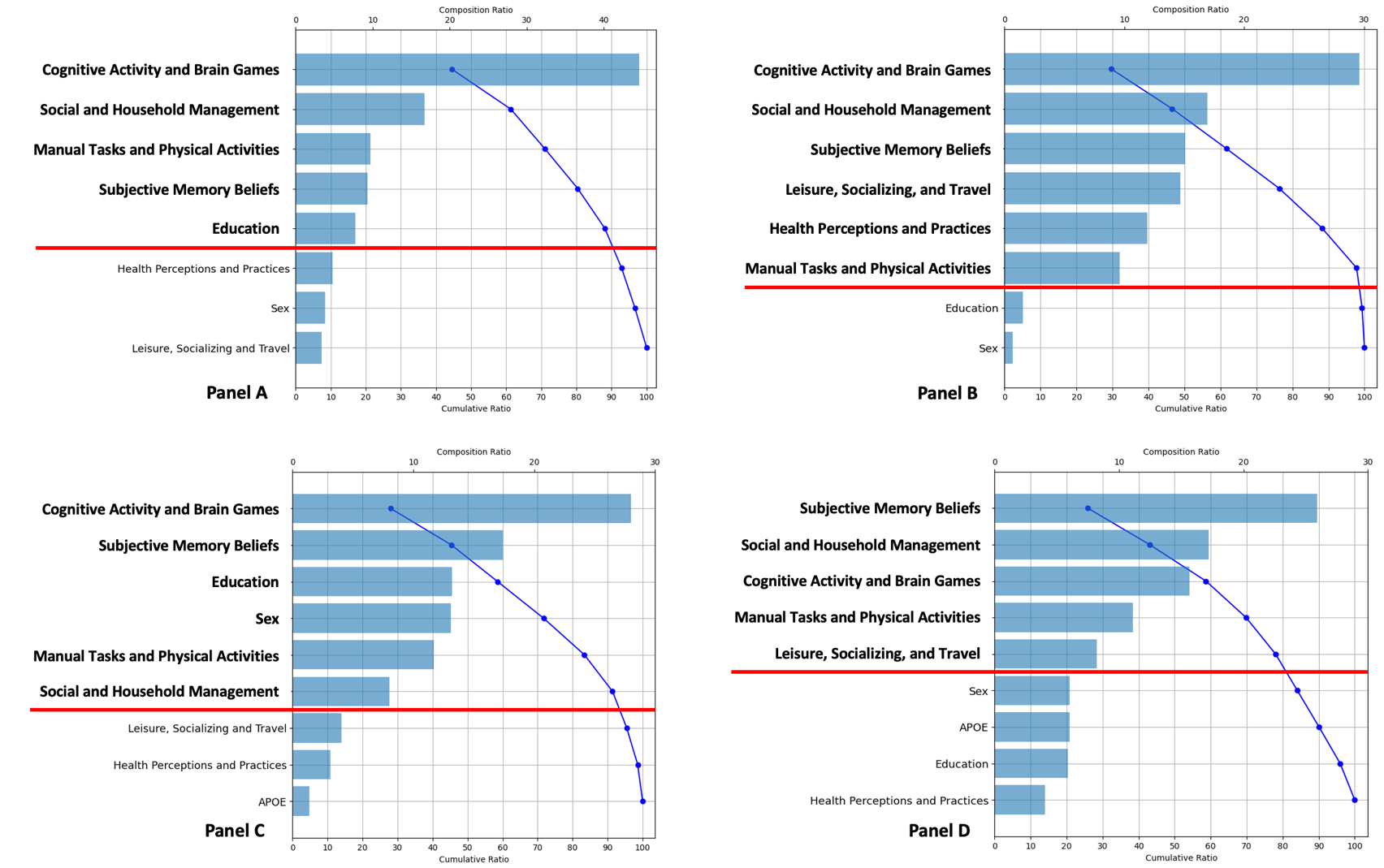
**

**Supplementary Figure 2.** Tree SHAP Waterfall Plots Depicting Results from Phase 1 (i.e., whole sample analyses; *N* = 746) and Phase 2 (i.e., genotyped subsample analyses; *n* = 562) of the Random Forest Analysis. Panel A: Relative importance of sex, gender facets, and education in discriminating the Low-Declining and High-Stable memory trajectory classes. Panel B: Relative importance of sex, gender facets, and education in discriminating the Low-Declining and Moderate/Normal-Declining memory trajectory classes. Panel C: Relative importance of sex, gender facets, education, and *Apolipoprotein E* (*APOE*) in discriminating the Low-Declining and High-Stable memory trajectory classes. Panel D: Relative importance of sex, gender facets, education, and *APOE* in discriminating the Low-Declining and Moderate/Normal-Declining memory trajectory classes. *APOE* was coded as an ordinal predictor representing successive increases in AD risk: 0 = ε2ε2 (least risk) and ε2ε3 (some protection); 1 = ε3ε3 (neutral risk); and 2 = ε2ε4 (some risk), ε3ε4 (more risk), and ε4ε4 (higher risk). In each panel, predictors are plotted in descending order of model contribution. The bars in each panel depict the individual composition ratio (i.e., the amount that each predictor contributes to the classification model; see top of panel for scale). The curved line represents the cumulative ratio (i.e., the total amount each successive predictor contributes to the model; see bottom of each panel for scale). Leading predictors are depicted in bold font above the red line.

**Supplementary References**

1. Dixon RA, de Frias CM. The Victoria Longitudinal Study: from characterizing cognitive aging to illustrating changes in memory compensation. Aging Neuropsychol Cogn. 2004;11(2-3):346–76.

2. Lezak MD. Neuropsychological assessment. New York, NY: Oxford University Press; 1983.

3. Vakil E, Blachstein H. Rey Auditory-Verbal Learning Test: structure analysis. J Clin Psychol. 1993;49(6):883-90.

4. Fah H, Bohn L, Greiner R, Dixon RA. Comparing machine learning classifier models in discriminating cognitively unimpaired older adults from three clinical cohorts in the Alzheimer’s disease spectrum: demonstration analyses in the COMPASS-ND study. Front Aging Neurosci. 2025; 17(1542514): 1-19.

5. Bohn L, Drouin SM, McFall GP, Rolfson DB, Andrew MK, Dixon RA. Machine learning analyses identify multi-modal frailty factors that selectively discriminate four cohorts in the Alzheimer’s disease spectrum: a COMPASS-ND study. BMC Geriatr. 2023;23(837):1-18.

6. McFall GP, Bohn L, Gee M, Drouin SM, Fah H, Han W et al. Identifying key multi-modal predictors of incipient dementia in Parkinson’s disease: a machine learning analysis and Tree SHAP interpretation. Front Aging Neurosci. 2023;15(1124232):1-16.

7. Drouin SM, McFall GP, Potvin O, Bellec P, Masellis M, Duchesne S, Dixon RA. Data-driven analyses of longitudinal hippocampal imaging trajectories: discrimination and biomarker prediction of change classes. J Alzheimers Dis. 2022;88(1):97-115.

8. McFall GP, McDermott KL, Dixon RA. Modifiable risk factors discriminate memory trajectories in non-demented aging: precision factors and targets for promoting healthier brain aging and preventing dementia. J Alzheimers Dis. 2019;70(s1):S101-18.

9. Python Software Foundation. Python 3.10.11 [Internet]. Python Software Foundation; 2023 Apr 5 [cited 2026 Jan 17]. Available from: https://www.python.org/downloads/release/python-31011/

10. Pedregosa F, Varoquaux G, Gramfort A, Michel V, Thirion B, Grisel O, et al. Scikit-learn: machine learning in Python. J Mach Learn Res. 2011;12:2825-30.
